# Laparoscopic versus open pancreaticoduodenectomy for pancreatic and periampullary tumor: A meta-analysis of randomized controlled trials and non-randomized comparative studies

**DOI:** 10.3389/fonc.2022.1093395

**Published:** 2023-01-25

**Authors:** Yong Yan, Yinggang Hua, Cheng Chang, Xuanjin Zhu, Yanhua Sha, Bailin Wang

**Affiliations:** ^1^ Department of General Surgery, Guangzhou Red Cross Hospital, Jinan University, Guangzhou, China; ^2^ Department of Laboratory Medicine, The Second Affiliated Hospital of Guangzhou University of Chinese Medicine, Guangzhou, China

**Keywords:** laparoscopic pancreaticoduodenectomy, open pancreaticoduodenectomy, whipple, pancreatic head, periampullary tumor, meta-analysis

## Abstract

**Objective:**

This meta-analysis compares the perioperative outcomes of laparoscopic pancreaticoduodenectomy (LPD) to those of open pancreaticoduodenectomy (OPD) for pancreatic and periampullary tumors.

**Background:**

LPD has been increasingly applied in the treatment of pancreatic and periampullary tumors. However, the perioperative outcomes of LPD versus OPD are still controversial.

**Methods:**

PubMed, Web of Science, EMBASE, and the Cochrane Library were searched to identify randomized controlled trials (RCTs) and non-randomized comparative trials (NRCTs) comparing LPD versus OPD for pancreatic and periampullary tumors. The main outcomes were mortality, morbidity, serious complications, and hospital stay. The secondary outcomes were operative time, blood loss, transfusion, postoperative pancreatic fistula (POPF), postpancreatectomy hemorrhage (PPH), bile leak (BL), delayed gastric emptying (DGE), lymph nodes harvested, R0 resection, reoperation, and readmission. RCTs were evaluated by the Cochrane risk-of-bias tool. NRCTs were assessed using a modified tool from the Methodological Index for Non-randomized Studies. Data were pooled as odds ratio (OR) or mean difference (MD). This study was registered at PROSPERO (CRD42022338832).

**Results:**

Four RCTs and 35 NRCTs concerning a total of 40,230 patients (4,262 LPD and 35,968 OPD) were included. Meta-analyses showed no significant differences in mortality (OR 0.91, *p* = 0.35), serious complications (OR 0.97, *p* = 0.74), POPF (OR 0.93, *p* = 0.29), PPH (OR 1.10, *p* = 0.42), BL (OR 1.28, *p* = 0.22), harvested lymph nodes (MD 0.66, *p* = 0.09), reoperation (OR 1.10, *p* = 0.41), and readmission (OR 0.95, *p* = 0.46) between LPD and OPD. Operative time was significantly longer for LPD (MD 85.59 min, *p* < 0.00001), whereas overall morbidity (OR 0.80, *p* < 0.00001), hospital stay (MD −2.32 days, *p* < 0.00001), blood loss (MD −173.84 ml, *p* < 0.00001), transfusion (OR 0.62, *p* = 0.0002), and DGE (OR 0.78, *p* = 0.002) were reduced for LPD. The R0 rate was higher for LPD (OR 1.25, *p* = 0.001).

**Conclusions:**

LPD is associated with non-inferior short-term surgical outcomes and oncologic adequacy compared to OPD when performed by experienced surgeons at large centers. LPD may result in reduced overall morbidity, blood loss, transfusion, and DGE, but longer operative time. Further RCTs should address the potential advantages of LPD over OPD.

**Systematic review registration:**

PROSPERO, identifier CRD42022338832.

## Introduction

Pancreaticoduodenectomy (PD) is the standard surgical treatment for pancreatic and periampullary tumors, which involves resection of the duodenum as well as with or without the distal stomach, the first portion of the jejunum, the pancreatic head, and common bile duct with the gallbladder (1). Owing to the complicated operation process and potentially lethal complications, PD should be performed by proficient surgeons in high-volume centers (2). Despite improved proficiency and technique, the perioperative mortality rate of PD remains up to 3% in highly specialized centers (3). PD remains the most difficult procedure of abdominal surgery and is associated with a high risk of morbidity and mortality (4).

The advantage of minimal invasiveness, reduced pain, and fast recovery from laparoscopic surgery has made it become the standard procedure in appendectomy and cholecystectomy (5, 6). With the accumulation of laparoscopic surgical experience and the development of surgical instruments, laparoscopic surgery has also been widely used in complex procedures such as gastrectomy, colectomy, and rectectomy, and it has been proven to be safe and feasible as compared with the open approach (7–9). During the past decades, there has been an increasing effort to perform more complex procedures like PD with a laparoscopic approach. However, a laparoscopic procedure in such complex conditions often arouses concerns with the difficult maneuvering of vascular structures, complicated digestive tract reconstruction, and prolonged operative time.

Laparoscopic pancreatoduodenectomy (LPD) was first reported in Canada in 1994 by Gagner et al., where it was performed with a pylorus-preserving procedure for a patient with chronic pancreatitis (10). In the initial stage, most surgeons choose to carry out LPD for patients with benign diseases. Due to improvements in surgical technique, LPD has been increasingly applied in the treatment of pancreatic and periampullary tumors, regardless of their benign or malignant nature (3). In recent years, a series of cohort studies have demonstrated the technical feasibility and safety of LPD for pancreatic and periampullary tumors (11, 12). However, the effects of LPD versus open pancreaticoduodenectomy (OPD) on morbidity, oncologic adequacy, and mortality are still controversial (13). Despite that there were several meta-analyses that assessed surgical and oncologic outcomes between LPD and OPD in the past 5 years (14–19), which included few articles and patients, and there had been conflicting pooled results. Some have included a few available randomized controlled trials (RCTs), which could not show any significant differences (14–16). Furthermore, some meta-analyses included studies that reported overlapped data from the same database during the crossed time period and therefore were prone to bias (17, 19–21). There is evidence that pooling of high-quality non-randomized comparative trials (NRCTs) is appropriate in a meta-analysis comparing surgical procedures, as existing RCTs are few, have a small sample size, and are underpowered (22).

This meta-analysis aims to assess the potential advantage of LPD compared with OPD for pancreatic and periampullary tumors using all the available high-quality published trials, RCTs and NRCTs, while focusing on perioperative outcomes such as mortality, morbidity, hospital stay, operative time, blood loss, transfusion, oncologic adequacy, reoperation, and readmission.

## Methods

This meta-analysis followed the Cochrane Handbook for Systematic Reviews of Interventions, as well as Preferred Reporting Items for Systematic Reviews and Meta-Analyses (PRISMA) recommendations (23, 24). This study was registered at PROSPERO (CRD42022338832).

### Search strategy

We searched the English-language literature published up to May 2022 in PubMed, Web of Science, EMBASE, and the Cochrane Library. The search terms were as follows: [laparoscopic OR laparoscopy OR laparoscopically assisted OR minimally invasive OR minimal invasive surgery] AND [open OR conventional OR open conventional surgery] AND [pancreatoduodenectomy OR pancreaticoduodenectomy OR Pancreatectomy OR Whipple] AND [randomized controlled trial OR prospective study OR comparative study OR retrospective study]. Two authors (Yong Yan and Yinggang Hua) independently conducted the literature search and a cross-check.

### Inclusion and exclusion criteria

Two authors (Yong Yan and Yinggang Hua) screened eligible articles independently according to the inclusion criteria: 1) the interventions compared included LPD versus OPD, 2) elective PD for pancreatic or periampullary tumor, 3) adult patients, and 4) reported perioperative outcomes. The exclusion criteria were as follows: 1) experimental or animal studies, 2) single surgical technique with no comparative data, 3) studies without perioperative data, 4) emergency PD for abdominal trauma, and 5) the publication type was editorial, abstract, letter, case report, and expert opinion. If papers had overlapping data, the latest studies with the biggest sample size were included.

### Data extraction

The data were extracted using a predefined data extraction sheet. Both authors (Yong Yan and Yinggang Hua) independently extracted data and then cross-checked them. In case of inconsistencies, a third author (Cheng Chang) was consulted to reach a consensus. Parameters such as study characteristics, demographic characteristics, baseline characteristics, baseline matching, methodological quality, inclusion diseases, surgical details, surgeons’ experience, and open conversion were extracted. The primary outcomes were postoperative mortality, overall postoperative complications, severe postoperative complications, and length of hospital stay. Furthermore, the secondary outcomes comprised operative time, estimated blood loss, intraoperative blood transfusion, pancreas-specific complications (such as postoperative pancreatic fistula (POPF), postpancreatectomy hemorrhage (PPH), bile leak (BL), and delayed gastric emptying (DGE)), oncologic outcomes (such as harvested lymph nodes and R0 resection), reoperation, and unplanned readmission.

### Quality assessment

The Cochrane risk-of-bias tool was used for assessing the methodological quality of all included RCTs (25). The Methodological Index for Non-randomized Studies (MINORS) is an instrument that was developed by a group of practicing surgeons in France and validated specifically for NRCT evaluation (26). In this meta-analysis, a modified scale from a previously published meta-analysis on a similar surgical comparison was adopted (27). In this modified MINORS score, four items were not included because they mainly evaluate the reporting quality and the validity of long-term outcomes. The score of sample size was assessed by the actual number of LPD cases, as follows: 0 points for less than 20 LPD cases, 1 point for 20 or more but less than 50 cases, and 2 points for 50 or more LPD cases. In total, eight items were evaluated, with a maximum score of 16 points. Studies with 12 or more points were considered high quality.

### Statistical analysis

Review Manager 5.4 was used to analyze the data. RCTs and NRCTs were first analyzed separately and then combined using a stratified analysis. For dichotomous data, the odds ratio (OR) with a 95% confidence interval (CI) was calculated. For continuous data, the mean difference (MD) with 95% CI was calculated. The median and range were converted to mean and standard deviation by using mathematical models of Hozo et al. (28), and the mean and standard deviation from the sample size, median, and interquartile range were estimated using mathematical models by Wan et al. (29) Heterogeneity was assessed by *I* ^2^, with values of 50% or more indicating significant heterogeneity. The random-effects model was used when *I* ^2^ was 50% or more, and the fixed-effects model was used when *I* ^2^ was less than 50%. *p* < 0.05 was statistically significant. Subgroup analysis and estimation of publication bias were also performed. Subgroup analysis was planned for studies of all malignancies, benign and malignant, LPD cases of 50 or less, LPD cases of more than 50, baseline matching incomplete, and baseline matching complete.

## Results

### Study selection

The flow diagram of the study selection procedure is shown in **Supplementary Figure 1**. The titles and abstracts of 1,651 studies were screened for inclusion. Of these, 1,591 were excluded due to ineligibility. The full texts of 60 studies were screened, of which three studies (30–32) presented patients from the National Cancer Database (NCDB) and two studies (33, 34) presented patients from the National Surgical Quality Improvement Program (NSQIP) with overlapped study intervals, and the latest studies (32, 34) with the biggest sample size were included. After assessment according to selection criteria and excluding overlapped studies, 39 studies published between 2012 and 2022 included in the quantitative synthesis were identified. These included 4 RCTs (35–38) and 35 NRCTs (32, 34, 39–71).

### Quality assessment

The summarized risk-of-bias assessment for RCTs is presented in **Supplementary Figure 2**. The random sequence generation was adequate in all RCTs. As the RCT of Poves et al. (36) did not specify the method of allocation concealment, it received an unclear risk-of-bias score. All included RCTs had a high risk of bias for blinding, except the RCT of van Hilst et al. (37) received a low risk-of-bias score for patient blinding by using a large abdominal dressing. All included RCTs had a low risk of attrition and reporting bias, but received a high risk of other bias, as the surgeons might not reach proficiency in three RCTs (35–37) and high cross-over in both groups might affect outcomes in RCT of Wang et al. (38) Assessment of NRCTs is displayed in **Supplementary Table 1**. The median score was 12 points. A total of 24 NRCTs with 12 or more points were considered high quality, whereas the remaining 11 NRCTs had scores between 10 and 11.

### Study characteristics

The major characteristics of included studies are summarized in **Table 1**. Four included RCTs were published after 2016 and carried out between 2013 and 2019 in four different countries (India, Spain, the Netherlands, and China). Two of the RCTs were multicentric studies (37, 38). The RCT of Palanivelu et al. (35) only included patients with periampullary cancers, while the other three RCTs (36–38) included patients with benign, premalignant, or malignant conditions. All RCTs were adequately matched in reviewed baseline characteristics. Of the 35 included NRCTs, 25 were conducted in three countries (10 in the USA, 8 in China, and 7 in South Korea). The study periods of included NRCTs mostly were between 2010 and 2020 (range 1993 to 2020), and 23 NRCTs were published between 2017 and 2022. All included NRCTs were retrospective studies, 15 of which were designed using propensity score matching analysis. A total of 18 NRCTs only included patients with malignant conditions, while the other 17 NRCTs included patients with benign, premalignant, and malignant conditions. The reported conversion rates in each included study varied between 3.1% and 24.1%.

**Table 1:** Study characteristics.

Study	Country	Study period	Study design	Included diseases	Conversion (%)	Matched factors	Not matched
Palanivelu 2017 (35)	India	2013–2015	Single-center, open-label, RCT	Periampullary cancers	3.1	1, 2, 3, 4, 5, 6, 7, 8	–
Poves 2018 (36)	Spain	2013–2017	Single-center, open-label, RCT	Benign, premalignant, malignant	23.5	1, 2, 3, 4, 5, 6, 7, 8	–
van Hilst 2019 (37)	Netherlands	2016–2017	Multicenter, patient-blinded, RCT	Benign, premalignant, malignant	20	1, 2, 3, 4, 5, 6, 7, 8	–
Wang M 2021 (38)	China	2018–2019	Multicenter, open-label, RCT	Benign, premalignant, malignant	3.7	1, 2, 3, 4, 5, 6, 7, 8	–
Ammori 2020 (39)	Jordan	2015–2018	Retrospective, PS	Benign or malignant	–	1, 2, 3, 6, 7, 8	–
Asbun 2012 (40)	USA	2005–2011	Retrospective, database	Benign, premalignant, malignant	–	1, 2, 3, 4, 5, 6, 7, 8	–
Chen K 2021 (41)	China	2004–2020	Retrospective, database, PS	Pancreatic ductal adenocarcinoma	9.9	1, 2, 3, 4, 5, 7, 8	–
Chen XM 2018 (42)	China	2013–2017	Retrospective	Periampullary malignancy	–	1, 2, 3, 4, 5, 7, 8	–
Choi 2020 (43)	South Korea	2014–2019	Retrospective	Pancreatic head cancer	–	1, 2, 3, 4, 6, 7, 8	–
Chopinet 2018 (44)	France	2002–2014	Retrospective	Periampullary tumors	9.2	1, 2, 3, 5, 7	4
Conrad 2017 (45)	USA	2000–2010	Retrospective, database	Periampullary or pancreatic head adenocarcinoma	–	1, 2, 3, 4, 7, 8	–
Croome 2014 (46)	USA	2008–2013	Retrospective, database	Pancreatic ductal adenocarcinoma	6.5	1, 3, 5, 7, 8	2
Dang C 2021 (47)	China	2011–2019	Retrospective, PS	Non-pancreatic periampullary adenocarcinoma	–	1, 2, 3, 5, 7, 8	–
Delitto 2016 (48)	USA	2010–2014	Retrospective, database	Periampullary malignancy	9.1	1, 2, 3, 5, 7	8
Ding W 2021 (49)	China	2015–2020	Retrospective, PS	Benign or malignant	–	1, 2, 3, 5, 7, 8	–
Dokmak 2014 (50)	France	2011–2014	Retrospective, database	Periampullary tumors	6.5	1, 2, 5, 7, 8	3
El Nakeeb 2020 (51)	Egypt	2013–2018	Retrospective, PS	Periampullary tumors	10.8	1, 2, 3, 5, 8	–
Han SH 2020 (52)	South Korea	2012–2017	Retrospective, PS	Benign, premalignant, malignant	–	1, 2, 3, 4, 5, 6, 8	–
Kantor 2017 (32)	USA	2010–2013	Retrospective, NCDB	Pancreatic adenocarcinoma		1, 7	–
Katsuki 2021 (53)	Japan	2016–2018	Retrospective, database	Benign or borderline tumors	–	1, 2, 5, 7	–
Khaled 2018 (54)	UK	2002–2015	Retrospective, PS	Malignant tumors	6.7	1, 2, 3, 4, 7, 8	–
Kuesters 2018 (55)	Germany	2010–2016	Retrospective, database	Pancreatic ductal adenocarcinoma	–	1, 2, 3, 7, 8	–
Lee CS 2018 (56)	South Korea	1993–2017	Retrospective, PS	Benign or borderline malignant	–	1, 2, 3, 4, 7, 8	–
Mazzola 2021 (57)	Italy	2013–2020	Retrospective, PS	Benign, premalignant, malignant	3.8	1, 2, 3, 4, 5, 7	–
Mendoza 2015 (58)	South Korea	2014	Retrospective	Periampullary tumors	–	1, 2, 3, 4, 5, 6, 8	–
Meng LW 2018 (59)	China	2010–2015	Retrospective, database	Non-pancreatic periampullary adenocarcinoma	–	1, 2, 3, 4, 5, 7, 8	–
Mesleh 2013 (60)	USA	2009–2012	Retrospective	Benign, premalignant, malignant	4	2, 3, 4, 7	–
Senthilnathan 2015 (61)	India	2006–2011	Retrospective, database	Periampullary and pancreatic head malignancy	–	1, 2, 3, 7, 8	–
Shin 2019 (62)	South Korea	2014–2017	Retrospective, PS	Periampullary malignancy	–	1, 2, 3, 4, 7, 8	–
Song KB 2015 (63)	South Korea	2007–2012	Retrospective, PS	Benign or low-grade malignant	–	1, 2, 3, 5, 6, 7, 8	
Stauffler 2016 (64)	USA	1995–2014	Retrospective, database	Pancreatic ductal adenocarcinoma	24.1	1, 2, 3, 5, 7	8
Tan CL 2015 (65)	China	2009–2014	Retrospective, database	Benign, premalignant, malignant	–	1, 2, 4, 5, 7	–
Tan JKH 2019 (66)	Singapore	2014–2016	Retrospective, PS	Malignant tumors	5	1, 2, 5, 8	
Tee MC 2015 (67)	USA	2007–2014	Retrospective, database	Benign, premalignant, malignant	–	1, 3, 5, 6, 7	2
Tran 2016 (68)	USA	2000–2010	Retrospective, NIS	Benign, premalignant, malignant	–	2, 5	1
Xourafas 2018 (34)	USA	2014–2016	Retrospective, NSQIP	Benign, premalignant, malignant	21	1, 2, 3, 4	5, 7
Yoo 2020 (69)	South Korea	2011–2017	Retrospective, PS	Ampulla of Vater cancer	–	1, 2, 3, 4, 5, 7, 8	–
Zhang Z 2022 (70)	China	2014–2018	Retrospective, PS	Pancreatic ductal adenocarcinoma	4.3	1, 2, 3, 4, 5, 6, 7, 8	–
Zhou W 2019 (71)	China	2013–2017	Retrospective, PS	Pancreatic ductal adenocarcinoma	–	1, 2, 3, 4, 5, 7	–

Matching factors: 1, age; 2, gender; 3, BMI; 4, ASA score; 5, comorbidities; 6, classic/pylorus-preserving PD; 7, malignancy rate; 8, tumor size.

BMI, body mass index; RCT, randomized controlled trial; PS, propensity score matching; ASA, American Society of Anesthesiologists; PD, pancreaticoduodenectomy; NCDB, National Cancer Database; NSQIP, National Surgical Quality Improvement Program; NIS, Nationwide Inpatient Sample.

The baseline characteristics of patients in each treatment group are summarized in **Table 2**. A total of 40,230 patients were included in the analysis with 4,262 (10.6%) having undergone LPD and 35,968 (89.4%) having undergone OPD, and 23,186 (57.6%) were women. Among them, 818 (2.0%) cases were from the four included RCTs, 411 (9.6% of all LPD cases) of which were in the LPD group and 407 in the OPD group (1.1% of all OPD cases). However, in 17 studies, 14 NRCTs and 3 RCTs had LPD cases of no more than 50. The mean/median age and body mass index (BMI) in each treatment group ranged from 49.6 to 76.5 years and 21.3 to 28.7 kg/m^2^, respectively. The overall comorbidity rates reported in each treatment group were between 37.6% and 61.2%. The most common comorbidities, diabetes mellitus (DM) and hypertension (HTN), ranged from 3.0% to 44.0% and 10.0% to 69.0%, respectively. The malignancy rate ranged from 66.4% to 97.3% in studies not specific to malignant conditions. The mean/median size of the tumor ranged from 1.8 to 3.6 cm.

**Table 2:** Demographic and baseline characteristics of patients in included studies.

Study	Procedure	Cases	Age (years)	No. (M/F)	BMI (kg/m^2^)	ASA I/II/III	Comorbidities, n (%)	Classic/pylorus-preserving PD	Malignancy, n (%)	Tumor size (cm)
Palanivelu 2017 (35)	LPD	32	57.8 ± 2.0	18/14	24.9 ± 0.7	13/17/2	15 (46.9)	10/22	32 (100)	3.3 ± 0.7
	OPD	32	58.6 ± 2.1	22/10	22.4 ± 0.6	11/18/3	17 (53.1)	6/26	32 (100)	3.6 ± 1.9
Poves 2018 (36)	LPD	32	69 (34–86)^e^	13/19	24 (16–33)^e^	1/18/13	5 (0–8)^k^	6/25	24 (75.0)	2.4 (0.9–7.0)^b^
	OPD	29	70 (36–83)^e^	20/9	26 (17–43)^e^	1/13/15	5 (1–10)^k^	11/18	25 (86.2)	2.9 (1.2–7.5)^b^
van Hilst 2019 (37)	LPD	50	67 (59–76)^a^	20/30	25 ± 3	5/32/13	DM 6 (12)	8/38	44 (80.0)	2.6 ± 14
	OPD	49	66 (61–73)^a^	25/24	26 ± 4	7/26/16	DM 10 (20)	8/39	43 (87.8)	2.6 ± 12
Wang M 2021 (38)	LPD	297	61 (54–67)^a^	171/126	22.4 ± 2.9	51/177/69	121 (41)	242/55	229 (77.1)	2.4 ± 0.7
	OPD	297	60 (52–66)^a^	193/104	22.1 ± 3.1	50/191/56	133 (45)	254/43	232 (78.1)	2.5 ± 0.9
Ammori 2020 (39)	LPD	11	57 (48–72)^b^	5/6	25.0 (17.0–44.9)^b^	–	–	11/0	9 (81.8)	3 (0–5)^b^
	OPD	22	63 (22–75)^b^	12/10	28.7 (15.5–39.4)^b^	–	–	14/8	18 (81.8)	3 (0–7.5)^b^
Asbun 2012 (40)	LPD	53	62.9 ± 14.14	29/24	27.64 ± 7.16	13/39/1^f^	DM 14 (26.4); HTN 30 (56.6)	3/39	51 (96.2)	2.74 ± 1.6
	OPD	215	67.3 ± 11.53	95/120	26.6 ± 5.08	37/163/13^f^	DM 60 (27.9); HTN 130 (60.5)	70/98	195 (90.6)	3.14 ± 1.5
Chen K 2021 (41)	LPD	101	62.4 ± 8.2	67/34	22.3 ± 2.5	47/52/2	44 (43.5)	–	101 (100)	3.0 ± 0.9
	OPD	101	62.2 ± 8.4	67/34	22.5 ± 2.6	45/54/2	46 (45.5)	–	101 (100)	3.1 ± 1.0
Chen XM 2018 (42)	LPD	47	63 ± 12	26/21	24 ± 3	1.5 ± 1^g^	DM 14 (29.8); HTN 14 (29.8)	–	47 (100)	2.5 ± 1.5
	OPD	55	66 ± 15	34/21	22.7 ± 3.3	1.5 ± 1^g^	DM 20 (36.3); HTN 18 (32.7)	–	55 (100)	3.0 ± 1.8
Choi 2020 (43)	LPD	27	63.3 ± 9.4	12/15	23.2 ± 2.1	2/12/13	–	0/27	27 (100)	2.8 ± 1.0
	OPD	34	63.3 ± 9.4	18/16	22.9 ± 3.4	2/16/16	–	0/34	34 (100)	2.9 ± 1.3
Chopinet 2018 (44)	LPD	65	62.6 (31–83)^b^	39/26	23 (15–35)^b^	52 (82)^h^	DM 13 (20); HTN 22 (34)	–	51 (78.5)	–
	OPD	290	62.7 (19–84)^b^	165/125	23 (17–38)^b^	210 (72)^h^	DM 45 (16); HTN 88 (30)	–	237 (81.7)	–
Conrad 2017 (45)	LPD	40	68 (45–83)^b^	26/14	23.9 (14.8–34.1)^b^	2 (1–3)^i^	–	–	40 (100)	2.5 (0.3–8.0)^b^
	OPD	25	66 (43–76)^b^	18/7	24.5 (20.7–31.1)^b^	2 (1–4)^i^	–	–	25 (100)	3.0 (1.3–6.0)^b^
Croome 2014 (46)	LPD	108	66.6 ± 9.6	51/57	27.4 ± 5.4	–	DM 33 (30.6); HTN 54 (50.0)	–	108 (100)	3.3 ± 1.0
	OPD	214	65.4 ± 10.9	131/83	27.2 ± 5.3	–	DM 67 (31.3); HTN 91 (42.5)	–	214 (100)	3.3 ± 1.3
Dang C 2021 (47)	LPD	131	57.4 ± 9.4	79/52	21.7 ± 2.8	–	DM 6 (4.5)	–	131 (100)	1.7 (1.3–2.5)^a^
	OPD	131	57.5 ± 9.5	81/50	21.6 ± 2.9	–	DM 4 (3.0)	–	131 (100)	1.7 (1.3–2.8)^a^
Delitto 2016 (48)	LPD	52	65.3^c^	34/18	26.3^c^	–	5.8^l^	–	52 (100)	2.5^c^
	OPD	50	68.6^c^	28/22	25.5^c^	–	6.0^l^	–	50 (100)	3.1^c^
Ding W 2021 (49)	LPD	112	66.0 ± 9.2	60/52	22.9 ± 2.7	–	DM 12 (10.7); HTN 35 (31.3)	–	84 (75.0)	2.9 ± 1.5
	OPD	112	64.1 ± 10.6	65/47	22.1 ± 3.9	–	DM 19 (20.0); HTN 32 (28.6)	–	90 (80.3)	3.2 ± 1.7
Dokmak 2015 (50)	LPD	46	60 (27–85)^b^	26/20	22.6 (17–30)^b^	–	DM 12 (26); HTN 11 (24)	–	36 (78.3)	2.82 (1.2–4)^b^
	OPD	46	63 (47–81)^b^	28/18	26.4 (19–42)^b^	–	DM 17 (39); HTN 17 (37)	–	36 (78.3)	2.51 (1.5–4)^b^
El Nakeeb 2020 (51)	LPD	37	54 (33–62)^b^	22/15	–	–	DM 11 (29.7); HTN 9 (24.3)	–	33 (89.2)	3 (1–4)^b^
	OPD	74	53 (17–63)^b^	40/34	–	–	DM 25 (33.8); HTN 17 (23)	–	62 (83.8)	3 (1–6)^b^
Han SH 2020 (52)	LPD	104	61.5 ± 12.0	53/51	23.57 ± 2.72	9/57/38	DM 33 (31.7); HTN 37 (35.6)	0/104	–	2.5 ± 1.3
	OPD	113	64.5 ± 9.2	70/43	23.22 ± 3.09	5/55/53	DM 42 (37.2); HTN 49 (43.4)	0/113	–	2.7 ± 1.2
Kantor 2017 (32)	LPD	828	65.9 ± 10.7	–	–	–	–	–	828 (100)	–
	OPD	7,385	65.7 ± 10.4	–	–	–	–	–	7,385 (100)	–
Katsuki 2021 (53)	LPD	95	61 (51–72)^a^	49/46	–	–	DM 42 (44); HTN 57 (60)	–	–	–
	OPD	380	63 (56–73)^a^	193/187	–	–	DM 167 (44); HTN 239 (63)	–	–	–
Khaled 2018 (54)	LPD	15	65 (35–78)^b^	8/7	23.4 (18–26)^b^	15 (100)^h^	–	–	15 (100)	2.0 (0.7–8.0)^b^
	OPD	15	64.3 (45–76)^b^	8/7	24.9 (22–28)^b^	15 (100)^h^	–	–	15 (100)	2.2 (1.5–7.8)^b^
Kuesters 2018 (55)	LPD	62	71^d^	31/31	24.7 (15–39)^b^	–	–	–	62 (100)	2.8 (0.1–7.5)^b^
	OPD	278	68^d^	137/141	24.7 (16–46)^b^	–	–	–	278 (100)	2.7 (0.3–13.0)^b^
Lee CS 2018 (56)	LPD	31	56.4 ± 14.6	14/17	24.0 ± 3.3	13/13/5	–	–	–	3.3 ± 2.1
	OPD	31	58.0 ± 11.4	13/18	23.8 ± 2.7	2,015/11/5	–	–	–	3.3 ± 2.3
Mazzola 2021 (57)	LPD	50	67.5 (61–75)^a^	25/25	25 (23–28)^a^	5/41/4	5 (4–5)^m^	–	44 (88)	–
	OPD	50	71 (65–76)^a^	25/25	25.5 (22–28)^a^	4/38/8	5 (4–6)^m^	–	44 (88)	–
Mendoza 2015 (58)	LPD	18	63.7 ± 10.9	10/8	22.7 ± 3.1	18 (100)^h^	0.8 (0–2)^m^	2/16	–	2.9 ± 0.9
	OPD	34	68.4 ± 7.6	21/13	21.9 ± 3.0	29 (85.3)^h^	1.0 (0–3)^m^	11/23	–	3.1 ± 2.9
Meng LW 2018 (59)	LPD	58	59.9 ± 9.1	32/26	22.2 ± 2.9	28/30^j^	DM 4 (6.9); HTN 9 (15.5)	–	58 (100)	1.8 (1.5–2.6)^a^
	OPD	58	60.3 ± 8.6	34/24	22.9 ± 2.3	34/24^j^	DM 6 (10.3); HTN 6 (10.3)	–	58 (100)	2.0 (1.9–3.0)^a^
Mesleh 2013 (60)	LPD	75	–	43/32	–	–	–	–	73 (97.3)	–
	OPD	48	–	23/25	–	–	–	–	42 (87.5)	–
Senthilnathan 2015 (61)	LPD	45	54 ± 11.6	17/28	27.6^c^	–	–	–	45 (100)	2.8^c^
	OPD	118	56 ± 10.8	59/59	28.1^c^	–	–	–	118 (100)	3.1^c^
Shin 2019 (62)	LPD	56	74.8 ± 3.7	27/29	22.8 ± 2.6	2.1 ± 0.5	–	–	56 (100)	2.7 ± 1.2
	OPD	56	74.7 ± 3.5	25/31	22.6 ± 2.3	2.1 ± 0.4	–	–	56 (100)	2.6 ± 1.2
Song KB 2015 (63)	LPD	93	49.6 ± 13.4	47/46	22.8 ± 2.7	–	1.6 (0–3)^m^	0/93	–	3.1 ± 1.4
	OPD	93	50.1 ± 13	47/46	23.1 ± 2.5	–	1.5 (0–3)^m^	0/93	–	3.4 ± 2.1
Stauffler 2016 (64)	LPD	58	69.9 (40.6–84.8)^b^	32/26	25.9 (17.7–49.6)^b^	–	DM 19 (32.8); HTN 33 (56.9)	–	53 (100)	2.5 (0.3–10.0)^b^
	OPD	193	68.9 (33.3–86.9)^b^	96/97	25.6 (15.0–46.1)^b^	–	DM 62 (32.1); HTN 128 (66.3)	–	198 (100)	3.5 (0.3–14.0)^b^
Tan CL 2015 (65)	LPD	30	59.3 ± 9.3	18/12	–	6/19/5	DM 3 (10.0); HTN 3 (10.0)	–	27 (90.0)	–
	OPD	30	59.9 ± 10.4	23/7	–	6/18/6	DM 2 (6.7); HTN 4 (13.3)	–	26 (86.7)	–
Tan JKH 2019 (66)	LPD	20	65 (37–82)^b^	11/9	–	–	12/7/1^n^	–	20 (100)	2.1 (1.0–3.5)^b^
	OPD	20	64 (46–84)^b^	11/9	–	–	12/6/2^n^	–	20 (100)	2.0 (1.0–4.0)^b^
Tee MC 2015 (67)	LPD	113	76.5 ± 4.3	51/62	26.9 ± 4.7	–	DM 29 (25.7); HTN 78 (69.0)	100/13	75 (66.4)	–
	OPD	225	76.4 ± 4.5	140/85	26.8 ± 4.3	–	DM 73 (32.4); HTN 154 (68.4)	195/30	192 (85.3)	–
Tran 2016 (68)	LPD	681	67 (58–73)^a^	377/304	–	–	417 (61.2)	–	–	–
	OPD	14,893	65 (56–73)^a^	7,701/7,192	–	–	8,744 (58.7)	–	–	–
Xourafas 2018 (34)	LPD	418	63 (19–87)^a^	233/185	27.6 (16–67)^a^	1/104/296	DM 94 (22); HTN 192 (46)	–	319 (76.3)	–
	OPD	9,963	65 (18–89)^a^	5,359/4,604	27.2 (15–69)^a^	37/2,239/7,006	DM 2,525 (25); HTN 5,305 (53)	–	8,020 (80.5)	–
Yoo 2020 (69)	LPD	69	62.8 ± 10.1	34/35	23.1 ± 2.7	5/56/8	2.2 ± 1.3^l^	–	69 (100)	1.9 ± 1.0
	OPD	69	63.2 ± 8.6	38/31	23.5 ± 3.3	7/55/7	2.3 ± 1.1^l^	–	69 (100)	1.8 ± 1.0
Zhang Z 2022 (70)	LPD	47	57.6 ± 8.3	31/16	21.3 ± 2.2	42 (89.4)^h^	DM 2 (4.3); HTN 6 (12.8)	47/0	47 (100)	2.6 ± 0.8
	OPD	47	57.5 ± 8.7	30/17	21.4 ± 2.6	41 (87.2)^h^	DM 3 (6.4); HTN 7 (14.9)	47/0	47 (100)	2.7 ± 1.0
Zhou W 2019 (71)	LPD	55	63 (54–69)^a^	40/15	23.0 (20.7–25.2)^a^	5/47/3	24 (43.6)	–	55 (100)	–
	OPD	93	64 (59–70.5)^a^	68/25	22.3 (20.3–23.9)^a^	18/73/2	35 (37.6)	–	93 (100)	–

LPD, laparoscopic pancreaticoduodenectomy; OPD, open pancreaticoduodenectomy; PD, pancreaticoduodenectomy; No. (M/F), number of patients (male/female); BMI, body mass index; ASA, American Society of Anesthesiologists; IQR, interquartile range; CCI, Charlson Comorbidity Index; DM, diabetes mellitus; HTN, hypertension.

a Median (IQR).

b Median (range).

c Mean.

d Median.

e Mean (range).

f ASA II/III/IV.

g Mean ± SD of ASA score.

h Number (%) of ASA score ≤II.

i Median (range) of ASA score.

j ASA II/III.

k Mean (range) of CCI.

l Mean ( ± SD) of CCI.

m Median (IQR) of CCI.

n CCI 0/1/2.

Eight NRCTs (34, 44, 46, 48, 50, 64, 67, 68) were not adequately matched in reviewed baseline characteristics such as age, gender, BMI, American Society of Anesthesiologists (ASA) score, comorbidities, malignancy rate, and tumor size. The tumors were larger in the OPD group than in the LPD group in the studies of Delitto et al. (48) and Stauffer et al. (64) There were more male patients in the OPD group than the LPD group in the studies of Croome et al. (46) and Tee et al. (67) Patients who have undergone LPD were slightly older than those who have undergone OPD in the study of Tran et al. (68) BMI was lower in the LPD group than the OPD group in the study of Dokmak et al. (50) Patients with ASA score of less than 3 were more in the LPD group than the OPD group in the study of Chopinet et al. (44) Malignant histologic subtypes and HTN were noted more in the OPD group in the study of Xourafas et al. (34) Although only malignances were included, pancreatic adenocarcinoma in the OPD group and ampullary adenocarcinoma in the LPD group were noted more in the study of Tan et al. (66)

### Primary outcomes

**Figure 1:**
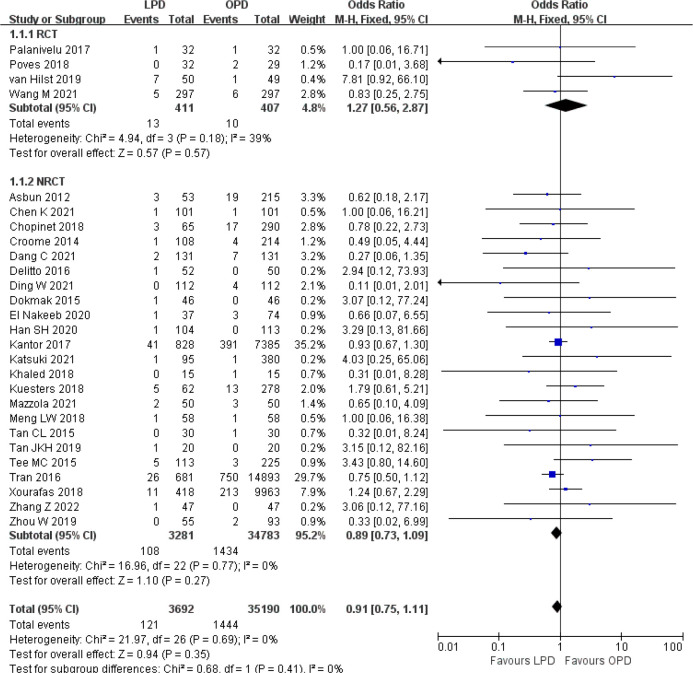
Forest plot of comparison between LPD and OPD on postoperative mortality. LPD, laparoscopic pancreaticoduodenectom; OPD, open pancreaticoduodenectomy.

**Figure 2:**
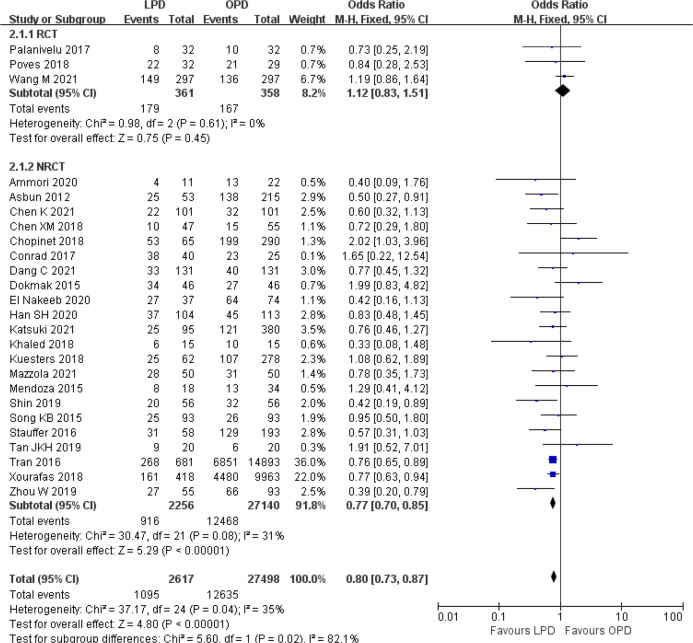
Forest plot of comparison between LPD and OPD on overall postoperative complications. LPD, laparoscopic pancreaticoduodenectom; OPD, open pancreaticoduodenectomy.

**Figure 3:**
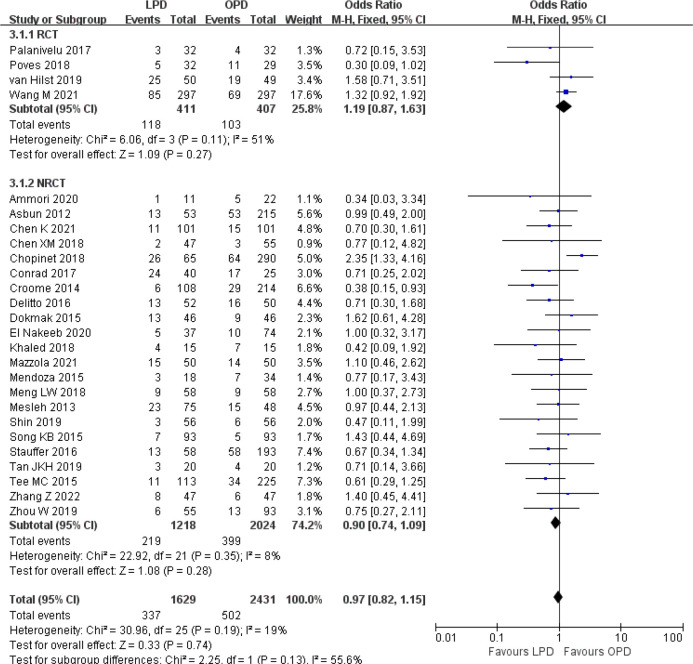
Forest plot of comparison between LPD and OPD on serious postoperative complications. LPD, laparoscopic pancreaticoduodenectom; OPD, open pancreaticoduodenectomy.

**Figure 4:**
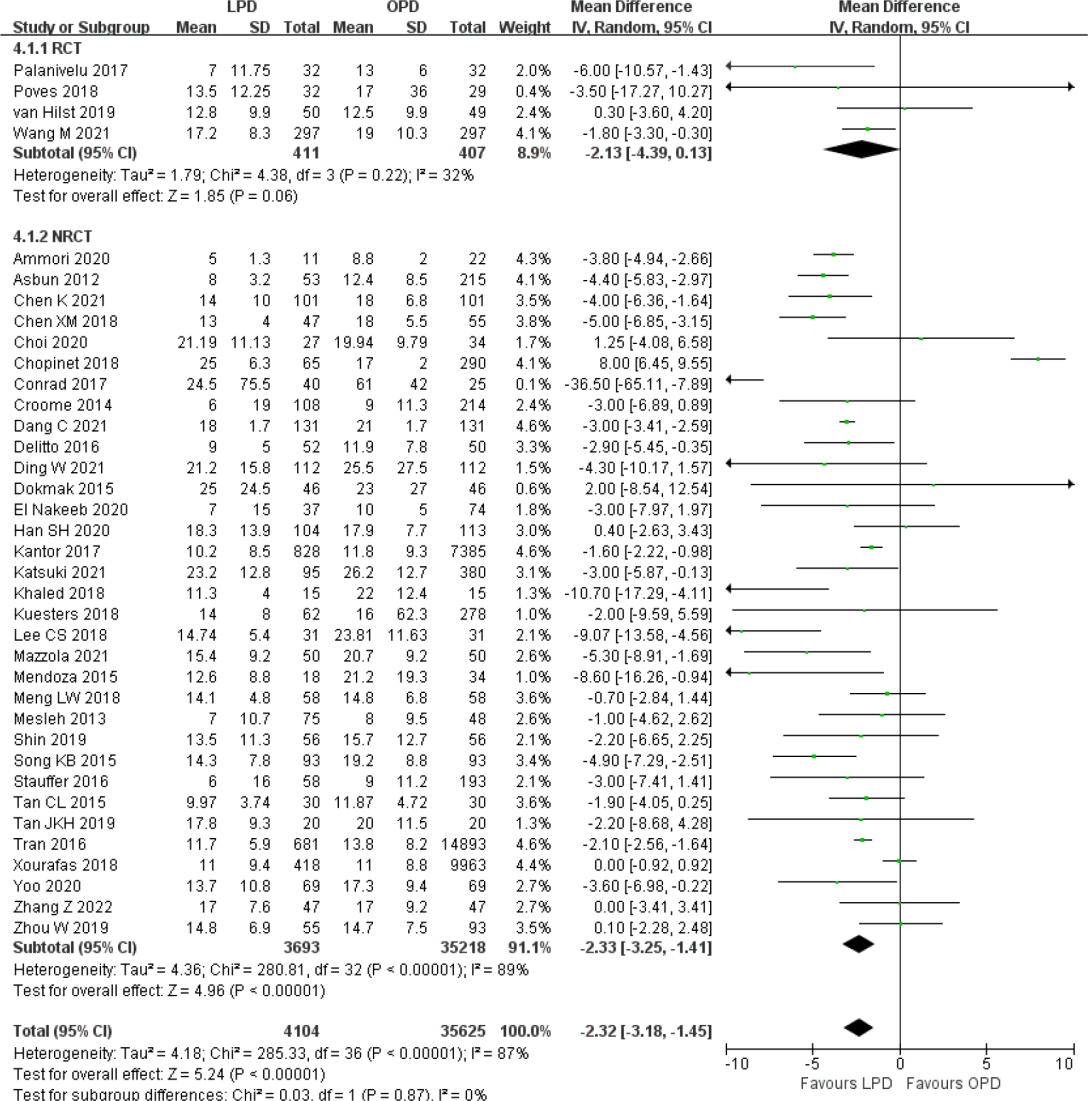
Forest plot of comparison between LPD and OPD on hospital stay. LPD, laparoscopic pancreaticoduodenectom; OPD, open pancreaticoduodenectomy.

There was no significant difference in postoperative mortality (OR 0.91, 95% CI 0.75–1.11, *p* = 0.35) (**Figure 1**). LPD was associated with a significant reduction in overall postoperative complications (OR 0.80, 95% CI 0.73–0.87, *p* < 0.00001) (**Figure 2**). However, serious postoperative complications were comparable (OR 0.97, 95% CI 0.82–1.15, *p* = 0.74) (**Figure 3**). There was significantly shorter hospital stay in the LPD group with significant heterogeneity (MD −2.32 days, 95% CI −3.18 to −1.45, *p* < 0.00001; *I* ^2^ = 87%, *p* < 0.00001); high heterogeneity was primarily observed in the NRCTs (*I* ^2^ = 89%, *p* < 0.00001) (**Figure 4**).

### Secondary outcomes

**Figure 5:**
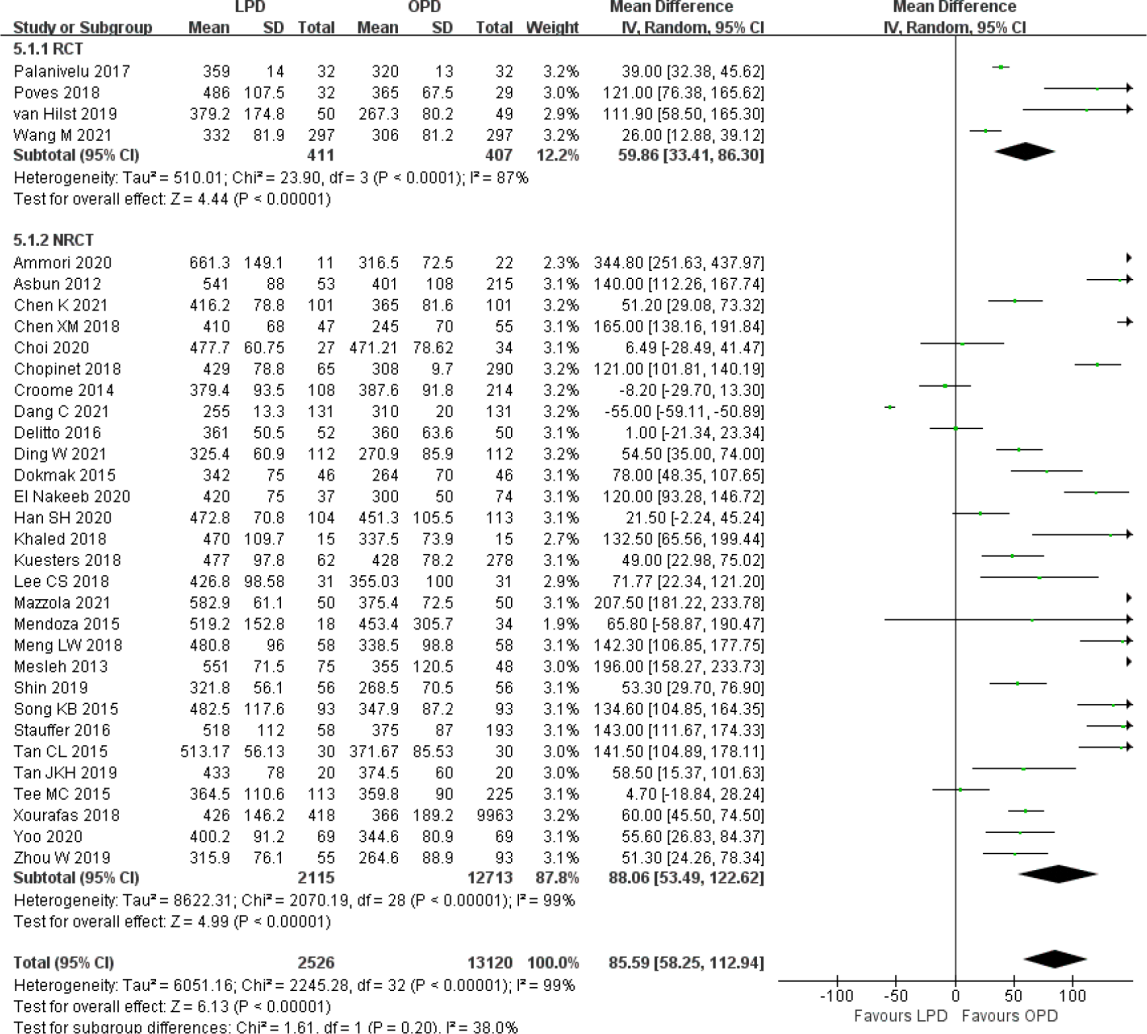
Forest plot of comparison between LPD and OPD on operative time. LPD, laparoscopic pancreaticoduodenectom; OPD, open pancreaticoduodenectomy.

**Figure 6:**
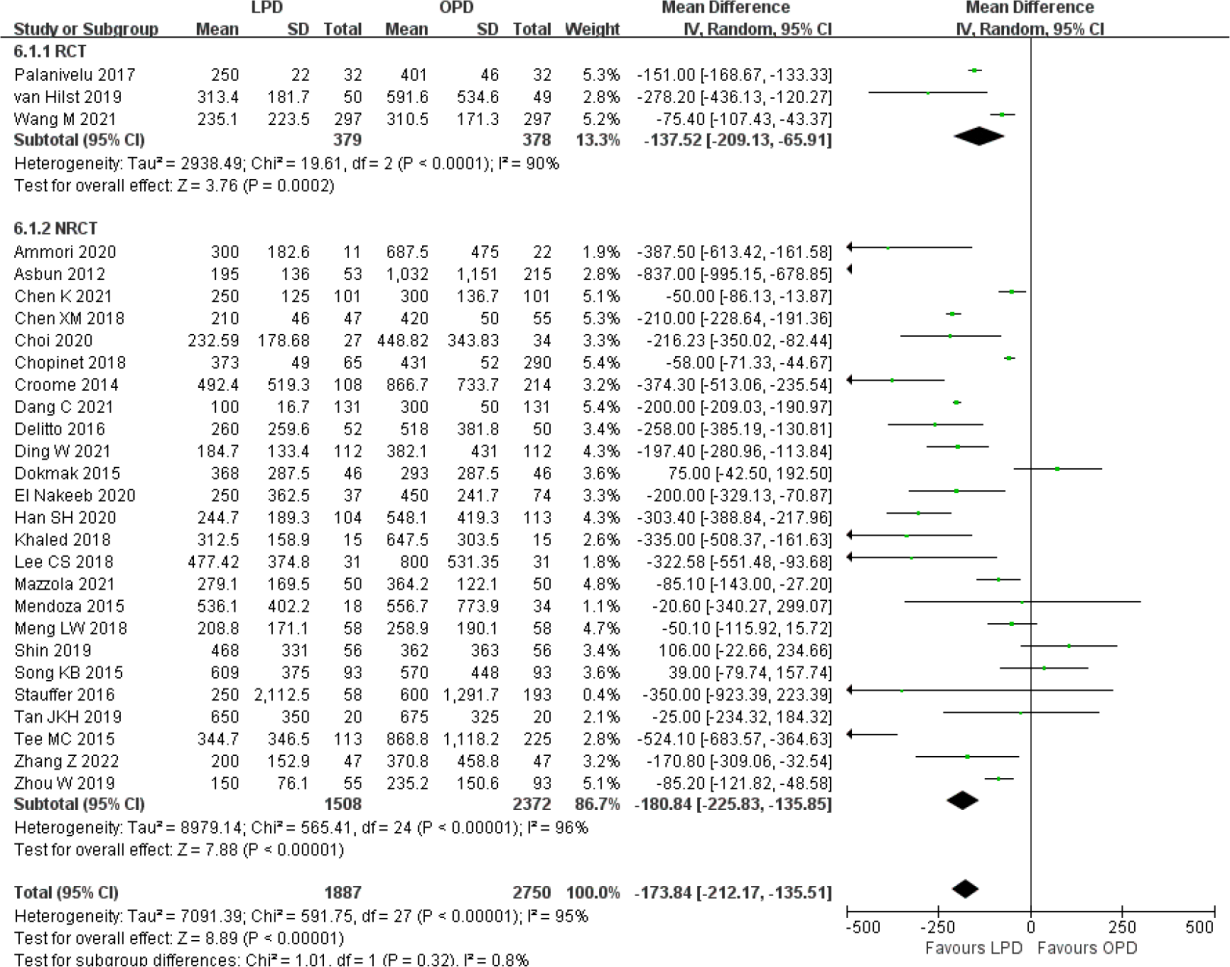
Forest plot of comparison between LPD and OPD on estimated blood loss. LPD, laparoscopic pancreaticoduodenectom; OPD, open pancreaticoduodenectomy.

**Figure 7:**
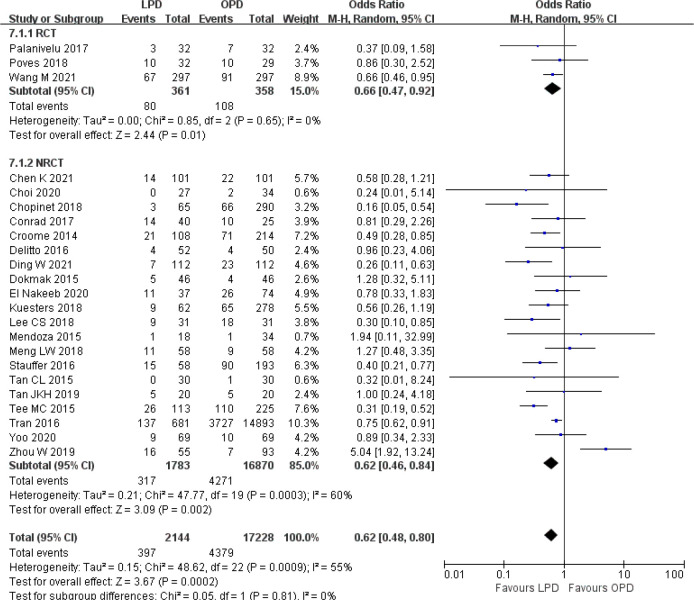
Forest plot of comparison between LPD and OPD on intraoperative blood transfusion. LPD, laparoscopic pancreaticoduodenectom; OPD, open pancreaticoduodenectomy.

There was significantly longer operative time in the LPD group with significant heterogeneity (MD 85.59 min, 95% CI 58.25–112.94, *p* < 0.00001; *I* ^2^ = 99%, *p* < 0.00001) (**Figure 5**). Estimated blood loss for LPD was less than that of OPD with significant heterogeneity (MD −173.84 ml, 95% CI −212.17 to −135.51, *p* < 0.00001; *I* ^2^ = 95%, *p* < 0.00001) (**Figure 6**). Significant heterogeneities were observed for operative time and estimated blood loss in both RCTs and NRCTs (*I* ^2^ = 87%, *p* < 0.0001; *I* ^2^ = 99%, *p* < 0.00001; *I* ^2^ = 90%, *p* < 0.0001; *I* ^2^ = 96%, *p* < 0.00001). In addition, LPD showed a significantly lower intraoperative blood transfusion rate than LPD with significant heterogeneity (OR 0.62, 95% CI 0.48–0.80, *p* = 0.0002; *I* ^2^ = 55%, *p* = 0.0009); heterogeneity was only observed in NRCTs (*I* ^2^ = 60%, *p* = 0.0003) (**Figure 7**).

**Figure 8:**
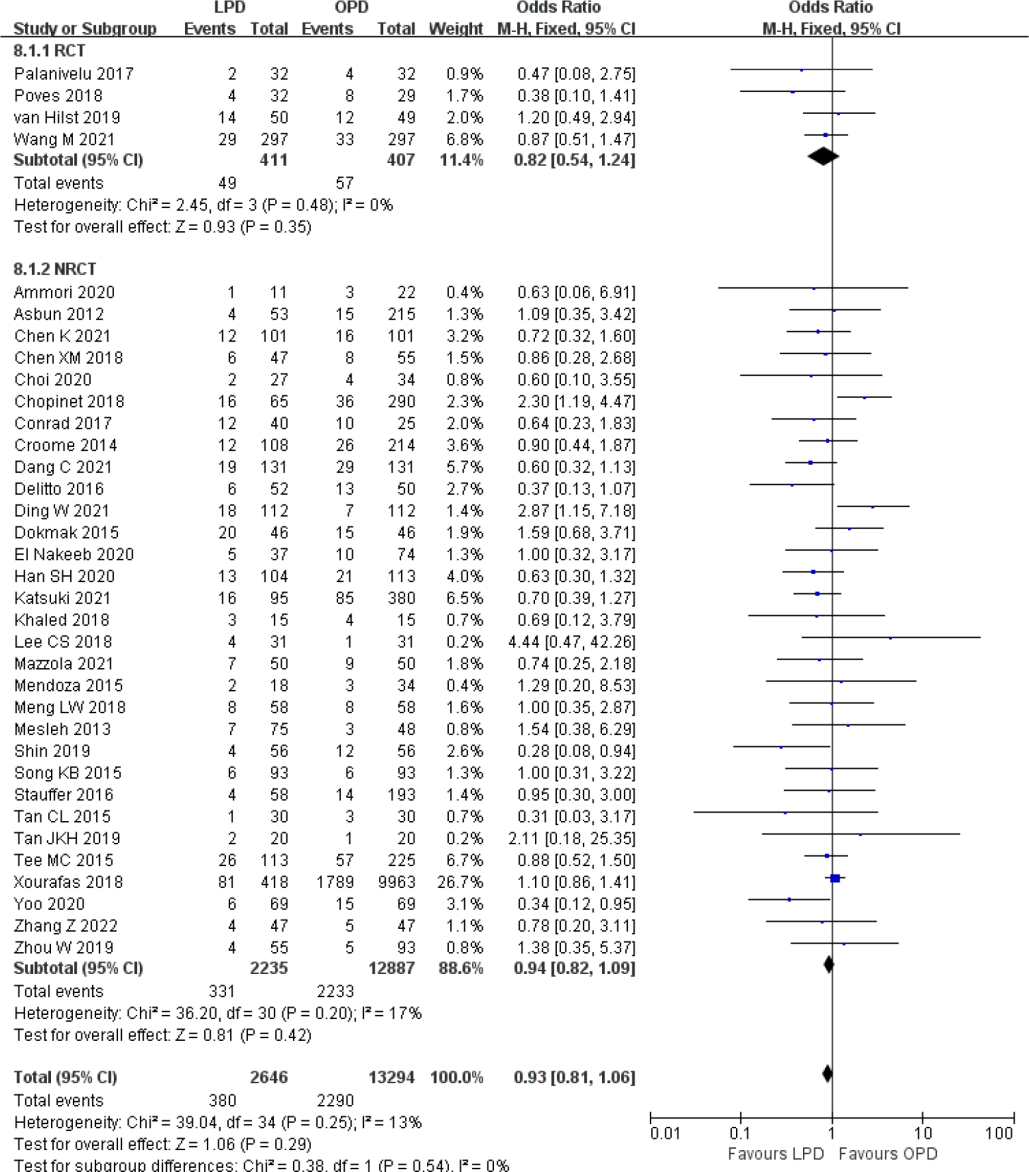
Forest plot of comparison between LPD and OPD on postoperative pancreatic fistula. LPD, laparoscopic pancreaticoduodenectom; OPD, open pancreaticoduodenectomy.

**Figure 9:**
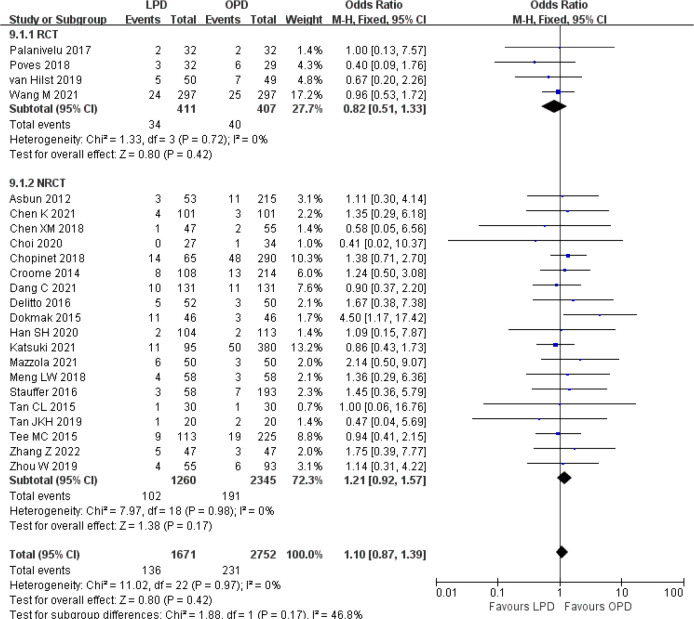
Forest plot of comparison between LPD and OPD on postpancreatectomy hemorrhage. LPD, laparoscopic pancreaticoduodenectom; OPD, open pancreaticoduodenectomy.

**Figure 10:**
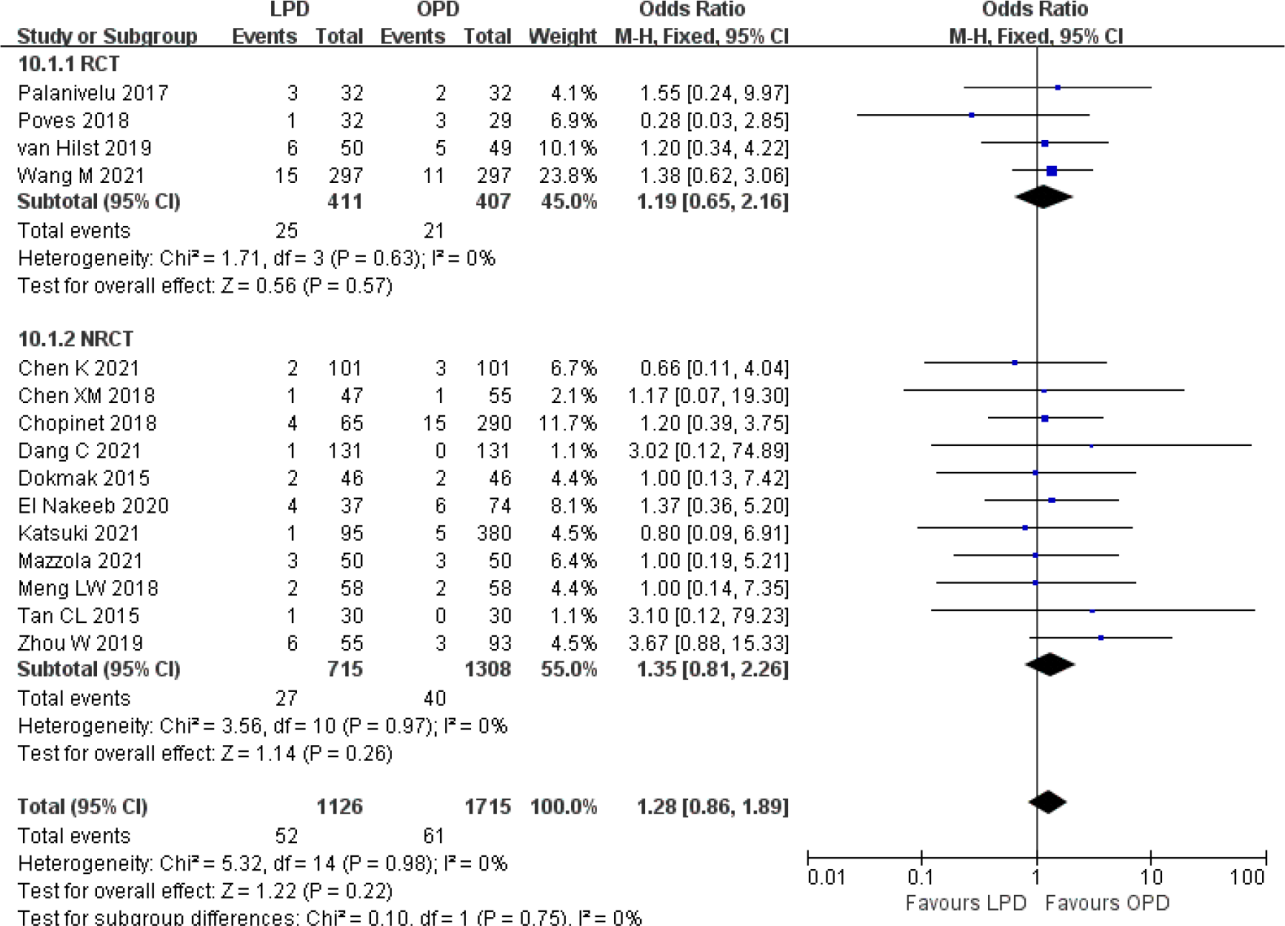
Forest plot of comparison between LPD and OPD on bile leak. LPD, laparoscopic pancreaticoduodenectom; OPD, open pancreaticoduodenectomy.

**Figure 11:**
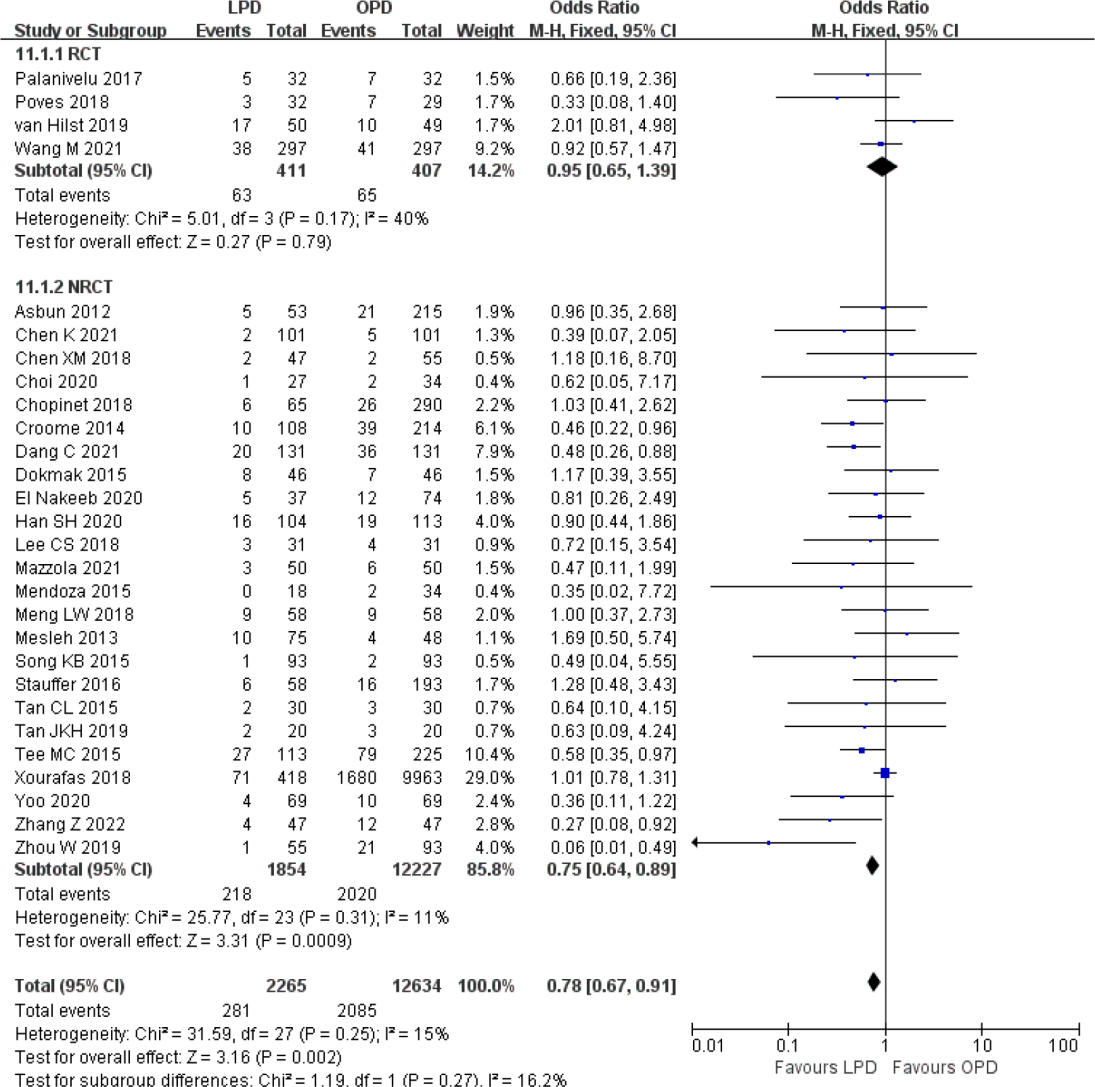
Forest plot of comparison between LPD and OPD on delayed gastric emptying. LPD, laparoscopic pancreaticoduodenectom; OPD, open pancreaticoduodenectomy.

**Figure 12:**
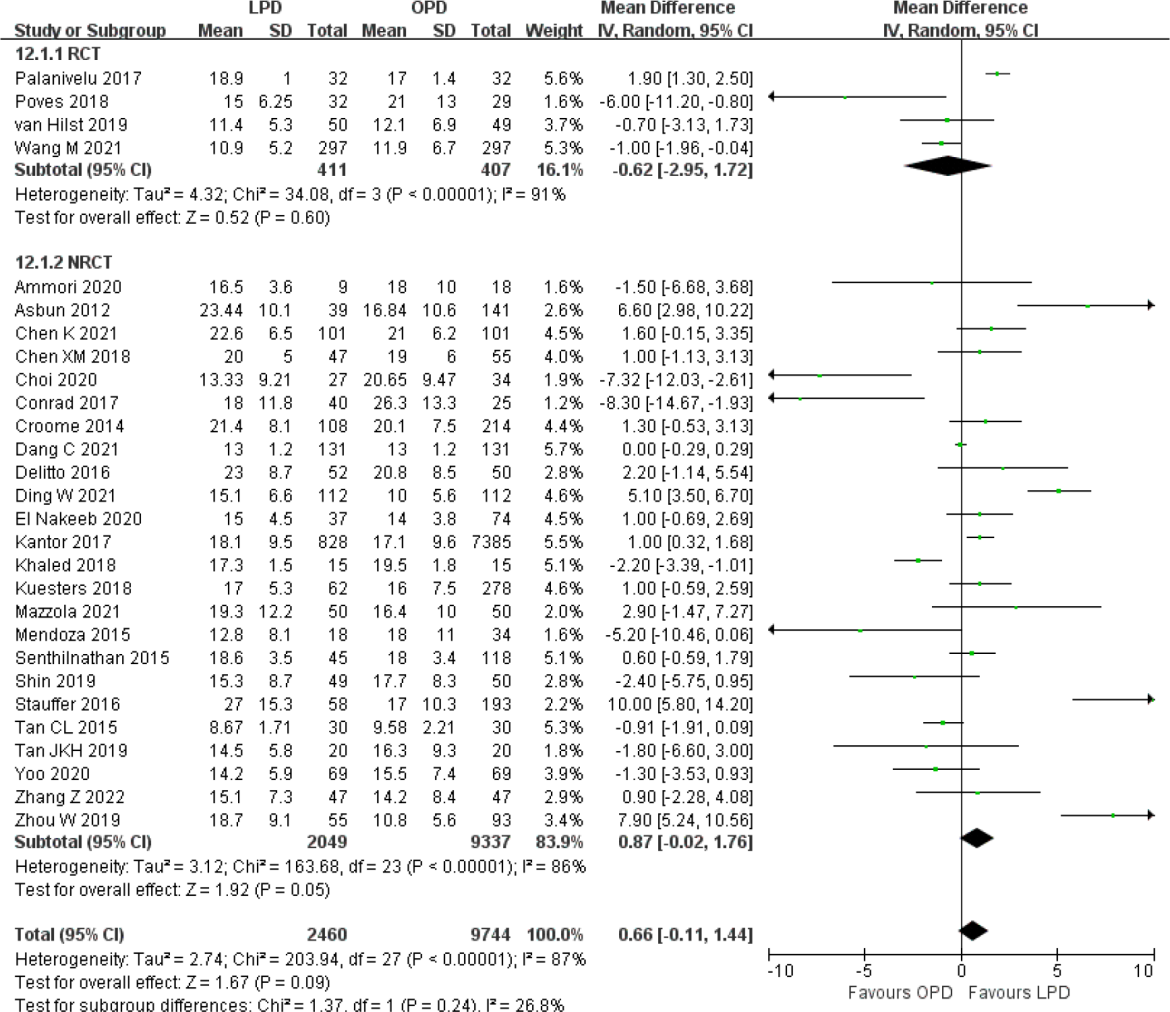
Forest plot of comparison between LPD and OPD on harvested lymph nodes. LPD, laparoscopic pancreaticoduodenectom; OPD, open pancreaticoduodenectomy.

**Figure 13:**
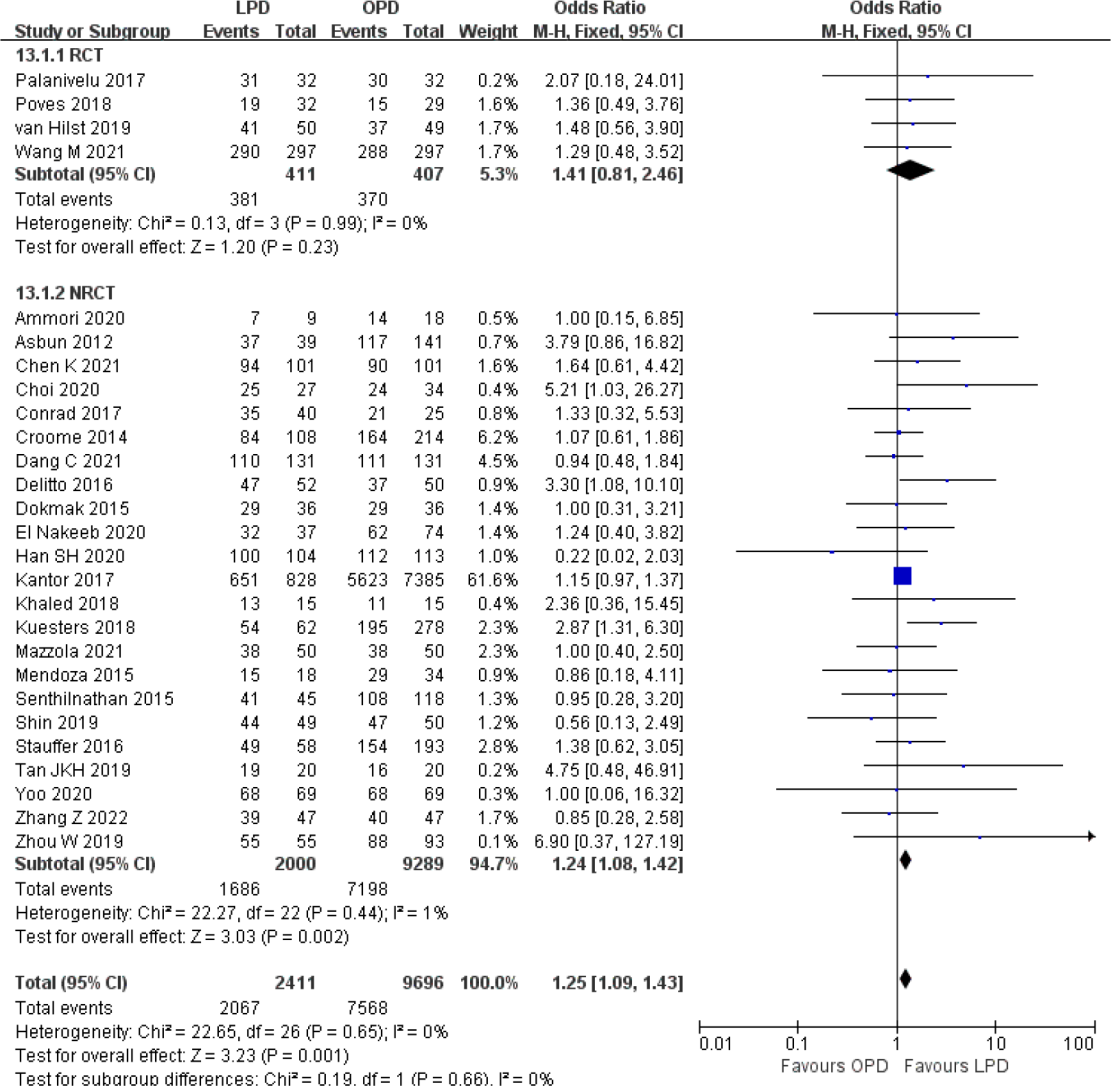
Forest plot of comparison between LPD and OPD on R0 resection. LPD, laparoscopic pancreaticoduodenectom; OPD, open pancreaticoduodenectomy.

There were no significant differences between LPD and OPD in terms of POPF (OR 0.93, 95% CI 0.81–1.06, *p* = 0.29), PPH (OR 1.10, 95% CI 0.87–1.39, *p* = 0.42), and BL (OR 1.28, 95% CI 0.86–1.89, *p* = 0.22) (**Figures 8**, **9**, **10**). However, a lower DGE rate was observed in the LPD group (OR 0.78, 95% CI 0.67–0.91, *p* = 0.002) (**Figure 11**). The number of harvested lymph nodes was similar in both groups with significant heterogeneity (MD 0.66, 95% CI −0.11–1.44, *p* = 0.09; *I* ^2^ = 87%, *p* < 0.00001), and heterogeneity was significant in both RCTs and NRCTs (*I* ^2^ = 91%, *p* < 0.00001; *I* ^2^ = 86%, *p* < 0.00001; respectively) (**Figure 12**). However, a higher R0 resection rate was found in the LPD group (OR 1.25, 95% CI 1.09–1.43, *p* = 0.001) (**Figure 13**). No significant differences in reoperation (OR 1.10, 95% CI 0.88–1.37, *p* = 0.41) and unplanned readmission (OR 0.95, 95% CI 0.81–1.10, *p* = 0.46) were found between LPD and OPD (**Supplementary Figures 3, 4**).

### Subgroup analysis and publication bias

Although the results of overall postoperative complications, hospital stay, DGE, and R0 resection were significantly different between LPD and OPD in the overall analysis, the subgroup analysis results showed consistent effects only in NRCTs and no significant differences in RCTs. However, the results of operative time, estimated blood loss, and intraoperative blood transfusion were significantly different in both RCTs and NRCTs.

The subgroup analysis results are partially summarized in **Supplementary Table 2**. The results of the postoperative mortality remained unchanged and had low heterogeneity among all subgroups evaluated. The reduction in overall postoperative complications was not significant when studies analyzed no more than 50 LPD cases (OR 0.85, 95% CI 0.61–1.18, *p* = 0.33) or baseline matching incomplete (OR 0.90, 95% CI 0.67–1.20, *p* = 0.46). The comparable result of serious postoperative complications favored LPD when studies analyzed all malignancies (OR 0.68, 95% CI 0.51–0.90, *p* = 0.008). Although heterogeneity remained high, the overall effect of hospital stay remained unchanged in the subgroup analysis, except that studies involved baseline matching incomplete, which became comparable between the two procedures (MD −0.16 days, 95% CI −3.18 to 2.87, *p* = 0.92).

The results of operative time and estimated blood loss remained unchanged and had high heterogeneity among all subgroups evaluated. The lower intraoperative blood transfusion rate became not significant when studies analyzed all malignancies (OR 0.76, 95% CI 0.50–1.16, *p* = 0.20) and no more than 50 LPD cases (OR 0.67, 95% CI 0.45–1.00, *p* = 0.05). A similar number of lymph nodes remained unchanged and had high heterogeneity among all subgroup analyses, except for subgroups of more than 50 LPD cases (MD 2.00, 95% CI 0.83–3.16, *p* = 0.0008), which favored LPD. Funnel plot analysis of the postoperative mortality and hospital stay indicates that the publication bias of these studies was not obvious (**Supplementary Figure 5**).

## Discussion

This meta-analysis compared the perioperative outcomes of LPD to OPD based on both RCTs and NRCTs, which included 4,262 patients who have undergone LPD. To our knowledge, it is the largest meta-analysis to date comparing LPD and OPD for pancreatic and periampullary tumors. From the perspective of pooled analysis results, this study supports the ongoing trend of laparoscopic surgery to cure tumors of the pancreatic head and periampullary region. Our findings clearly demonstrate that LPD leads to lower overall postoperative complications, hospital stay, estimated blood loss, intraoperative blood transfusion, and DGE, and it improves R0 resection rate with similar retrieval of lymph nodes. Rates of postoperative mortality, serious postoperative complications, POPF, PPH, BL, reoperation, and unplanned readmission are comparable between LPD and OPD. However, the duration of surgery is longer for patients undergoing LPD.

Published meta-analyses that only included RCTs were incomplete, and data from a small volume of cases prevented a valid comparison between LPD and OPD. The recent meta-analyses included three RCTs that did not show any difference between LPD and OPD except for longer operative time and lower blood loss in LPD (14–16). However, these findings were limited due to the high risk of bias and low certainty of evidence. As the surgeons in three RCTs (35–37) might not have surmounted the learning curve for LPD, the non-significant results pooled from RCTs did not mean that the LPD had no advantage over OPD, and these results should be interpreted with caution. Moreover, no subgroup analysis was performed in any of these studies due to the small number of patients and events included. In our analysis, more cases from NRCTs were analyzed. Quality assessment of the included NRCTs was based on a validated tool developed for NRCT evaluation, as well as 24 of which had a high-quality score and constituted two-thirds of the included studies. In consistent with these RCT meta-analyses, our analysis also showed LPD reduced blood loss and prolonged operative time. Although there was high heterogeneity within included studies and among subgroup analyses, it suggested a consistent difference. In addition, lower intraoperative blood transfusion was found in this meta-analysis, which had significant differences in both the RCT and NRCT subgroups.

Recently, several meta-analyses have been conducted comparing LPD to OPD for pancreatic ductal adenocarcinoma (17–19). The studies of Sun et al. (17) and Feng et al. (19) showed a higher R0 rate in LPD, whereas Chen et al. (18) found no significant difference between the techniques. The meta-analysis of Sun et al. (17) showed lower intraoperative blood transfusion in LPD, whereas Feng et al. (19) showed no difference. The meta-analysis of Sun et al. (17) just showed shorter hospital stay in LPD, while Feng et al. (19) further demonstrated less overall morbidity, serious postoperative complications, and estimated blood loss in LPD. However, the meta-analysis of Sun et al. (17) included a study about robotic PD (RPD), whereas Chen et al. (18) did not include two eligible studies of Kantor et al. (32) and Delitto et al. (48) Furthermore, both meta-analyses of Sun et al. (17) and Feng et al. (19) included two studies of Kantor et al. (32) and Chapman et al. (30), which reported overlapped patient data from the NCDB. One recent network meta-analysis from Kamarajah et al. (20) compared open, laparoscopic, and robotic PD for periampullary cancers and showed similar mortality, serious complications, POPF, BL, and R0 resection between LPD and OPD, but shorter hospital stay and higher retrieval of lymph nodes in LPD. Another similar network meta-analysis from Aiolfi et al. (21) compared open, laparoscopic, and robotic PD in the setting of the malignant, borderline, or benign disease and also showed similar mortality, serious complications, POPF, lymph nodes retrieved, and R0 resection between LPD and OPD, but reduced hospital stay, estimated blood loss, overall postoperative complications, and readmission in LPD. However, the two network analyses included studies of Kantor et al. (32), Chapman et al. (30), and Sharpe et al. (31), which reported overlapped data from NCDB, as well as studies of Xourafas et al. (34) and Zimmerman et al. (33), which presented overlapped data from NSQIP. Overall, these previous meta-analyses showed inconstant results and suffered some degree of bias. Compared to these previous studies, our analyses excluded the possible overlapped patient data and conducted a comparison specifically for LPD and OPD. Furthermore, our study performed subgroup analysis in terms of all malignancies, benign and malignant, LPD cases of 50 or less, LPD cases of more than 50, baseline matching incomplete, and baseline matching complete, which might add precision to our comparison of LPD versus OPD.

Our meta-analyses support the notion that LPD is equally safe as the conventional open approach. There was no significant difference regarding the rate of postoperative mortality, serious postoperative complications, POPF, PPH, BL, reoperation, and unplanned readmission. Over the last decade, increasing improvements in equipment and surgical technique have extended indications of LPD (1, 2). Despite these advances, perioperative mortality remains up to 3% for LPD in high-volume centers (3, 4). The postoperative mortality rates of LPD and OPD for pancreatic and periampullary tumors in our meta-analysis were comparable (3.3% and 4.1%, respectively). With no significant difference, serious postoperative complication rates for LPD and OPD in our meta-analysis were 20.7% and 20.6%, respectively. Pancreas-specific complications such as POPF, PPH, and BL are common morbidity after pancreatic surgery, which accounted for the main cause of surgical mortality (72–74). Severe POPF, PPH, or BL is a dreaded adverse event that may cause metabolic disorders, peritonitis, intraperitoneal empyema or abscess, anemia, sepsis, shock, and sometimes reoperation, as well as leads to fasting accompanying intravenous nutrition and prolonged hospital stay (72–74). Almost all of the included studies defined these complications according to the International Study Group of Pancreatic Surgery (ISGPS) (72–74), most of which reported clinically relevant grades (grades B and C). Consistent with the results of most existing clinical studies (35–38), the analyses of these complications for LPD and OPD in our meta-analysis were comparable and had high homogeneity within included studies. Overall, LPD has been shown to be comparable to OPD in the safety related to the operation for the treatment of pancreatic and periampullary tumors.

One striking finding for our meta-analysis was decreased DGE in the LPD versus OPD. Although not imminently life-threatening, DGE remains the most common complication after PD, which causes significant discomfort and results in prolonged hospital stay (75). A review study assessed the average rate of clinically relevant DGE (grades B and C) after PD was 14.3% according to the ISGPS definition (76). Consistently, as most of the included studies reported clinically relevant DGE according to the ISGPS definition, DGE rates for LPD and OPD in our meta-analysis were 12.4% and 16.5%, respectively. The exact mechanisms of DGE are still unclear and are mostly multifactorial results involving vagal denervation. A recent retrospective study demonstrated that preservation of the hepatic branch of the vagus nerve could help reduce the incidence of DGE during LPD (77). These results are likely to be related to fine dissection and meticulous manipulation, as laparoscopic surgery offers a magnified view that facilitates precise identification of nerves and effective prevention of excessive nerve injury. A potential advantage of LPD is that reducing DGE incidence should be taken into account, and prospective studies are warranted to validate this effect in the future.

LPD is a more technically demanding and time-consuming procedure, which can be attributed to the challenging dissection of the pancreatic head and difficult reconstruction of the digestive tract by laparoscopic tools. Due to the complicated operation process, it is generally accepted that achieving a good level of surgical proficiency for LPD requires a long learning curve. Some studies have shown the number of cases required to surmount the learning curve ranged from 40 to 60 LPD cases (78). The included RCTs had reported their surgeons’ LPD experience when the trials started, which is all less than 40 LPD cases except for the RCT of Wang et al. (38) with at least 104 LPD cases. As is the case with all surgical studies, the surgeon’s technical proficiency plays an important role in the postoperative outcomes of LPD. Unfortunately, most included NRCTs did not explicitly describe their proficiency in LPD, which prevented subgroup analysis addressing relations between surgical proficiency and outcomes. Nevertheless, most of the included studies were carried out at large-volume hospitals, and therefore, LPD is likely not appropriate for low-volume hospitals (2, 4). We believe that LPD should only be implemented in high-volume centers by experienced specialists who have performed a sufficient amount of procedures and surmounted the learning curve for LPD.

With conducted analysis specifically for all malignancies, the comparable result of serious postoperative complications favored LPD. In addition, the pooled estimate showed a higher R0 rate for LPD. Given the higher surgical requirement for malignancy, surgeons’ selection bias could have influenced these results, and these results should be interpreted with caution. Surgeons might be more conservative and discreet to choose LPD for malignancy because lymphadenectomy and negative margin are indispensable for radical cure and can be more challenging during laparoscopic surgery; therefore, healthier patients with earlier tumor stage may have been chosen for LPD especially in the initial stage of performing LPD (34, 44, 48, 64, 79). The lower intraoperative blood transfusion rate became not significant in LPD when studies analyzed all malignancies, which could be attributed to the increased complexity of radical resection by the laparoscopic approach. As LPD for malignancy might attenuate the effect of lower blood loss and transfusion, surgical indication selection for LPD should be considered and explored in future studies.

The lower overall postoperative complications and intraoperative blood transfusion for LPD became not significant in the subgroup analysis of studies with no more than 50 LPD cases. Although the operative time was still longer for LPD than the open procedure, the subgroup analysis of studies with more than 50 LPD cases demonstrated a significant reduction of operative time in LPD compared to studies with no more than 50 LPD cases. Given the detection power of sample size, some studied outcomes such as postoperative morbidity, intraoperative blood transfusion, and operative time may also have been influenced by sample size issues (4, 14, 80). Although all RCTs explicitly described their calculation method for ascertaining sample size, the ascertained sample sizes had great discrepancy. The RCTs of Palanivelu et al. (35) and Poves et al. (36) calculated the sample size according to the primary outcome of hospital stay and indicated that only 32 patients were required in each group. However, Wang et al. (38) also calculated hospital stay and assessed that the minimum number of patients required in each group was 274. Furthermore, the RCT of van Hilst et al. (37) calculated a sample size of 68 patients for each group according to the time of postoperative functional recovery but was prematurely terminated with only 50 LPD cases. It was suggested that some of the current studies may be underpowered for comparing complex surgical procedures, and further trials with larger numbers of patients are indispensable to clarify surgical outcomes between LPD and OPD with adequate statistical power.

The improved overall postoperative complications and hospital stay were influenced by studies of baseline matching incomplete in subgroup analysis. We think outcomes such as hospital stay and overall complications are susceptible to bias, where comparable baseline characteristics and standard definitions are important for accurate comparison of surgical procedures. In addition, our analyses showed that serious postoperative complications were not reduced in the LPD group. Because the laparoscopic technique is less invasive, LPD might mainly decrease medical and minor surgical complications. This is not surprising because the reduced medical and minor surgical complications of the laparoscopic technique also could help to reduce hospital stay (27, 81). However, a more detailed analysis of postoperative complications was not performed due to lack of information. LPD could provide benefits in reducing medical and minor surgical complications and hospital stay; further studies designed to validate this phenomenon should match completely, unify perioperative management, and use standard outcome definition.

There were several limitations in this study. Significant heterogeneity was shown in some outcomes, which might be explained by differences in study design, sample size, surgeons’ proficiency, baseline characteristics, healthcare system, postoperative recovery protocol, and other factors. Variations in sample size among studies were large, and some studies enrolled patients during a wide study period, which may have introduced biases due to the advancement in mastering surgical skills and improvement in surgical instruments. Pilot studies that might be more prone to choose LPD for benign or low-grade malignant patients may also have introduced biases. All of the factors above might make the surgical results more susceptible to the methodological quality of clinical trials and lead to high heterogeneity among studies. The economical results and long-term oncologic outcomes are not evaluated in our study, as adequate data are missing at present. With the technological advances of computer vision and artificial intelligence playing a role in the improvement of LPD, whether these theoretical advantages could translate into improved patient outcomes, especially for a more complex condition such as malignancy, needs further studies (82, 83).

In conclusion, the results of our meta-analysis suggest that LPD is associated with non-inferior short-term surgical outcomes and oncologic adequacy compared to OPD when performed by experienced surgeons at high-volume centers. LPD may result in reduced overall postoperative morbidity, blood loss, intraoperative transfusion, and DGE, but prolonged operative time. In addition, recent studies have addressed the issue of surgical safety of LPD but may not have been sufficiently powered to evaluate the differences in postoperative complications between LPD and OPD. Further RCTs are required to investigate whether there are advantages of LPD for the management of pancreatic and periampullary tumors.

## Data availability statement

The original contributions presented in the study are included in the article/**Supplementary Material**. Further inquiries can be directed to the corresponding authors.

## Author contributions

Data acquisition: YY, YH, and YS. Manuscript drafting: YY, YH, and YS. Statistical analysis: YY, CC, and XZ. Manuscript revision: BW and YS. All authors contributed to the article and approved the submitted version.
