# Supplementary material for: Laparoscopic versus open pancreaticoduodenectomy for pancreatic and periampullary tumor: A meta-analysis of randomized controlled trials and non-randomized comparative studies
Source: Front Oncol. 2023 Jan 25;12:1093395. doi: 10.3389/fonc.2022.1093395 (PMC9905842; doi:10.3389/fonc.2022.1093395)
Supplement: Supplementary file 1 [file DataSheet_1.docx]

**Laparoscopic Versus Open Pancreaticoduodenectomy for Pancreatic and Periampullary Tumors:**

***A Meta-analysis of Randomized Controlled Trials and Nonrandomized Comparative Studies***

**Supplementary Table 1.** Modified MINORS Score of All Eligible Nonrandomized Comparative Studies

| Study | Consecutive Patients | Prospective Data Collection | Reported Endpoints | Unbiased Outcome Evaluation^*^ | Appropriate Controls | Contemporary Groups | Groups Equivalent^#^ | Sample Size | Score |
| --- | --- | --- | --- | --- | --- | --- | --- | --- | --- |
| Ammori 2020^35^ | 2 | 1 | 1 | 1 | 2 | 1 | 2 | 0 | 10 |
| Asbun 2012^36^ | 1 | 1 | 2 | 1 | 2 | 1 | 2 | 2 | 12 |
| Chen K 2021^37^ | 1 | 2 | 2 | 1 | 2 | 2 | 2 | 2 | 14 |
| Chen XM 2018^38^ | 1 | 1 | 2 | 1 | 2 | 2 | 2 | 1 | 12 |
| Choi 2020^39^ | 1 | 1 | 1 | 1 | 2 | 2 | 2 | 1 | 11 |
| Chopinet 2018^40^ | 2 | 1 | 2 | 1 | 1 | 2 | 1 | 2 | 12 |
| Conrad 2017^41^ | 2 | 2 | 2 | 0 | 1 | 2 | 1 | 1 | 11 |
| Croome 2014^42^ | 2 | 2 | 2 | 1 | 2 | 2 | 1 | 2 | 14 |
| Dang C 2021^43^ | 1 | 1 | 2 | 1 | 2 | 1 | 2 | 2 | 12 |
| Delitto 2016^44^ | 2 | 2 | 2 | 1 | 2 | 2 | 1 | 2 | 14 |
| Ding W 2021^45^ | 1 | 1 | 1 | 1 | 2 | 2 | 2 | 2 | 12 |
| Dokmak 2014^46^ | 2 | 2 | 2 | 1 | 2 | 2 | 1 | 1 | 13 |
| El Nakeeb 2020^47^ | 1 | 2 | 2 | 1 | 2 | 2 | 2 | 1 | 13 |
| Han SH 2020^48^ | 1 | 1 | 1 | 1 | 2 | 2 | 1 | 2 | 11 |
| Kantor 2017^49^ | 1 | 1 | 2 | 0 | 2 | 2 | 2 | 2 | 12 |
| Katsuki 2021^50^ | 1 | 1 | 2 | 0 | 2 | 2 | 2 | 2 | 12 |
| Khaled 2018^51^ | 1 | 2 | 1 | 1 | 2 | 2 | 2 | 0 | 11 |
| Kuesters 2018^52^ | 1 | 2 | 2 | 0 | 1 | 1 | 2 | 2 | 11 |
| Lee CS 2018^53^ | 1 | 1 | 1 | 1 | 2 | 1 | 2 | 1 | 10 |
| Mazzola 2021^54^ | 2 | 2 | 2 | 1 | 2 | 1 | 2 | 2 | 14 |
| Mendoza 2015^55^ | 1 | 2 | 1 | 1 | 2 | 2 | 2 | 0 | 11 |
| Meng LW 2018^56^ | 1 | 2 | 2 | 1 | 1 | 2 | 2 | 2 | 13 |
| Mesleh 2013^57^ | 2 | 2 | 1 | 1 | 2 | 2 | 2 | 1 | 13 |
| Senthilnathan 2015^58^ | 1 | 2 | 1 | 0 | 2 | 2 | 1 | 1 | 10 |
| Shin 2019^59^ | 1 | 1 | 1 | 1 | 2 | 2 | 2 | 2 | 12 |
| Song KB 2015^60^ | 2 | 1 | 2 | 1 | 2 | 2 | 1 | 2 | 13 |
| Stauffler 2016^61^ | 2 | 1 | 2 | 1 | 2 | 1 | 1 | 2 | 12 |
| Tan CL 2015^62^ | 2 | 1 | 1 | 1 | 2 | 2 | 2 | 1 | 12 |
| Tan JKH 2019^63^ | 1 | 2 | 1 | 1 | 2 | 2 | 2 | 2 | 13 |
| Tee MC 2015^64^ | 2 | 1 | 2 | 0 | 2 | 2 | 1 | 2 | 12 |
| Tran 2016^65^ | 1 | 1 | 1 | 0 | 2 | 2 | 1 | 2 | 10 |
| Xourafas 2018^66^ | 1 | 2 | 1 | 1 | 1 | 2 | 1 | 2 | 11 |
| Yoo 2020^67^ | 1 | 1 | 1 | 1 | 2 | 2 | 2 | 2 | 12 |
| Zhang Z 2022^68^ | 1 | 2 | 1 | 1 | 2 | 2 | 2 | 1 | 12 |
| Zhou W 2019^69^ | 2 | 1 | 2 | 1 | 2 | 2 | 2 | 2 | 14 |

^*^ Considered incomplete if there was no protocol for analgesic administration, resume of oral intake or hospital discharge.

^#^ Factors considered: age, gender, BMI, ASA score, comorbidities, classic/pylorus-preserving PD, malignancy rate, and tumor size.

MINORS indicates Methodological Index for Nonrandomized Studies; BMI, Body Mass Index; ASA, American Society of Anesthesiologists; PD, pancreaticoduodenectomy.

**Supplementary Table 2.** Subgroup Analysis for All Malignancy/Benign and Malignant, LPD Less/More than 50 Cases, and Baseline Matching Incomplete/Complete

| Subgroup/Outcome | No. Studies | No. LPD case | No. OPD case | OR/MD | 95% CI | *P* | *I*^2^ |
| --- | --- | --- | --- | --- | --- | --- | --- |
| Postoperative mortality | | | | | | | |
| All malignancy | 12 | 1509 | 8424 | 0.92 | 0.69, 1.24 | 0.60 | 0% |
| Benign and malignant | 15 | 2183 | 26766 | 0.9 | 0.69, 1.17 | 0.43 | 10% |
| ≤50 LPD cases | 10 | 359 | 392 | 1.23 | 0.60, 2.51 | 0.57 | 0% |
| ＞ 50 LPD cases | 17 | 3333 | 34798 | 0.89 | 0.72, 1.09 | 0.26 | 0% |
| Baseline matching incomplete | 7 | 1483 | 25681 | 0.91 | 0.67, 1.23 | 0.55 | 8% |
| Baseline matching complete | 20 | 2209 | 9509 | 0.91 | 0.70, 1.18 | 0.47 | 0% |
| Overall postoperative complications | | | | | | | |
| All malignancy | 11 | 617 | 999 | 0.67 | 0.53, 0.84 | 0.0006 | 11% |
| Benign and malignant | 14 | 2000 | 26499 | 0.82 | 0.74, 0.91 | 0.0002 | 44% |
| ≤50 LPD cases | 10 | 308 | 377 | 0.85 | 0.61, 1.18 | 0.33 | 12% |
| ＞ 50 LPD cases | 15 | 2309 | 27121 | 0.79 | 0.72, 0.87 | 0.00001 | 48% |
| Baseline matching incomplete | 5 | 1268 | 25385 | 0.9 | 0.67, 1.20 | 0.46 | 70% |
| Baseline matching complete | 20 | 1349 | 2113 | 0.8 | 0.69, 0.93 | 0.004 | 21% |
| Serious postoperative complications | | | | | | | |
| All malignancy | 13 | 689 | 959 | 0.68 | 0.51, 0.90 | 0.008 | 0% |
| Benign and malignant | 13 | 940 | 1472 | 1.18 | 0.96, 1.45 | 0.12 | 30% |
| ≤50 LPD cases | 13 | 445 | 498 | 0.93 | 0.67, 1.29 | 0.67 | 0% |
| ＞ 50 LPD cases | 13 | 1184 | 1933 | 0.99 | 0.81, 1.20 | 0.9 | 44% |
| Baseline matching incomplete | 6 | 442 | 1018 | 0.88 | 0.49, 1.60 | 0.68 | 72% |
| Baseline matching complete | 20 | 1187 | 1413 | 1 | 0.81, 1.22 | 0.97 | 0% |
| Length of hospital stay | | | | | | | |
| All malignancy | 18 | 1806 | 8856 | -2.61 | -3.52, -1.71 | 0.00001 | 64% |
| Benign and malignant | 19 | 2298 | 26769 | -2.13 | -3.69, -0.57 | 0.007 | 92% |
| ≤50 LPD cases | 16 | 533 | 593 | -3.72 | -5.25, -2.19 | 0.00001 | 58% |
| ＞ 50 LPD cases | 21 | 3571 | 35032 | -1.66 | -2.70, -0.62 | 0.002 | 91% |
| Baseline matching incomplete | 7 | 1428 | 25649 | -0.16 | -3.18, 2.87 | 0.92 | 96% |
| Baseline matching complete | 30 | 2676 | 9976 | -2.88 | -3.59, -2.16 | 0.00001 | 64% |
| Operative time | | | | | | | |
| All malignancy | 15 | 891 | 1399 | 57.65 | 20.85, 94.45 | 0.002 | 99% |
| Benign and malignant | 18 | 1635 | 11721 | 108.17 | 78.86, 137.48 | 0.00001 | 95% |
| ≤50 LPD cases | 14 | 446 | 521 | 116.04 | 76.06, 156.02 | 0.00001 | 96% |
| ＞ 50 LPD cases | 19 | 2080 | 12599 | 64.68 | 28.77, 100.60 | 0.0004 | 99% |
| Baseline matching incomplete | 7 | 860 | 10981 | 56.62 | 15.26, 97.97 | 0.007 | 96% |
| Baseline matching complete | 26 | 1666 | 2139 | 93.89 | 61.62, 126.16 | 0.00001 | 99% |
| Estimated blood loss | | | | | | | |
| All malignancy | 14 | 807 | 1099 | -147.55 | -186.69, -108.41 | 0.00001 | 92% |
| Benign and malignant | 14 | 1080 | 1651 | -211.48 | -284.46, -138.50 | 0.00001 | 93% |
| ≤50 LPD cases | 13 | 431 | 509 | -165.96 | -211.95, -119.96 | 0.00001 | 80% |
| ＞ 50 LPD cases | 15 | 1456 | 2241 | -183.16 | -241.77, -124.56 | 0.00001 | 97% |
| Baseline matching incomplete | 6 | 442 | 1018 | -227.33 | -397.35, -57.30 | 0.009 | 93% |
| Baseline matching complete | 22 | 1445 | 1732 | -164.38 | -201.74, -127.02 | 0.00001 | 93% |
| Intraoperative blood transfusions | | | | | | | |
| All malignancy | 12 | 682 | 1167 | 0.76 | 0.50, 1.16 | 0.2 | 55% |
| Benign and malignant | 11 | 1462 | 16061 | 0.52 | 0.37, 0.74 | 0.0002 | 58% |
| ≤50 LPD cases | 10 | 313 | 355 | 0.67 | 0.45, 1.00 | 0.05 | 0% |
| ＞ 50 LPD cases | 13 | 1831 | 16873 | 0.61 | 0.44, 0.84 | 0.003 | 72% |
| Baseline matching incomplete | 7 | 1123 | 15911 | 0.49 | 0.32, 0.76 | 0.001 | 70% |
| Baseline matching complete | 16 | 1021 | 1317 | 0.7 | 0.57, 0.87 | 0.001 | 47% |
| Lymph nodes harvested | | | | | | | |
| All malignancy | 18 | 1786 | 8910 | 0.73 | -0.17, 1.63 | 0.11 | 87% |
| Benign and malignant | 10 | 674 | 834 | 0.42 | -1.46, 2.29 | 0.66 | 87% |
| ≤50 LPD cases | 15 | 499 | 630 | -0.84 | -2.09, 0.41 | 0.19 | 83% |
| ＞ 50 LPD cases | 13 | 1961 | 9114 | 2 | 0.83, 3.16 | 0.0008 | 90% |
| Baseline matching incomplete | 3 | 218 | 457 | 4.2 | -0.44, 8.83 | 0.08 | 86% |
| Baseline matching complete | 25 | 2242 | 9287 | 0.38 | -0.42, 1.17 | 0.35 | 87% |

LPD, laparoscopic pancreaticoduodenectomy; OPD, open pancreaticoduodenectomy; OR, odds ratio; MD, mean difference; CI, confidence interval.


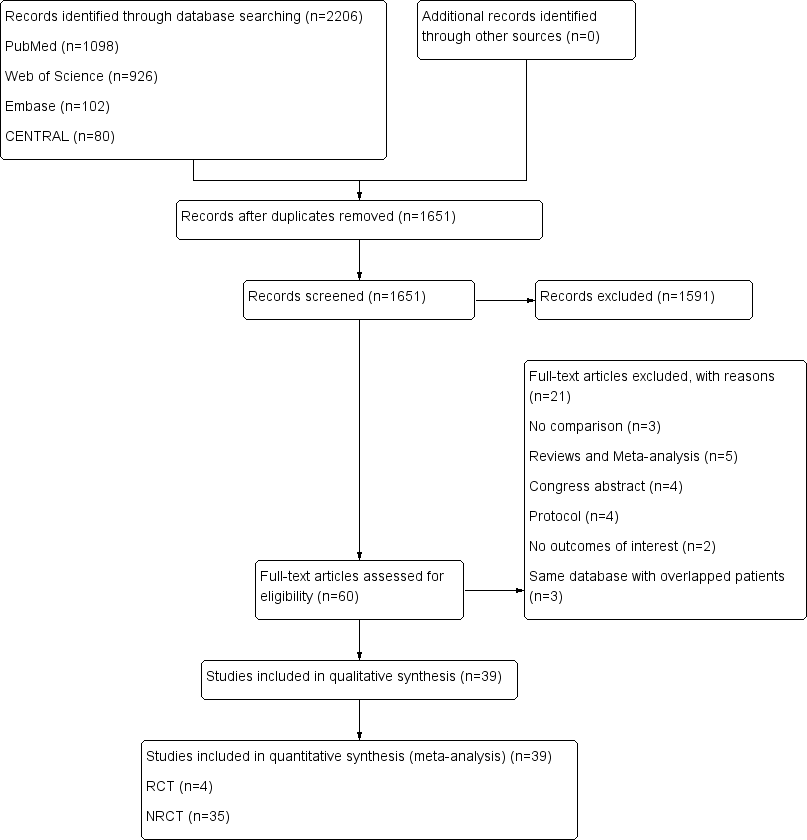


**Supplementary Figure 1.** PRISMA selection flow diagram.


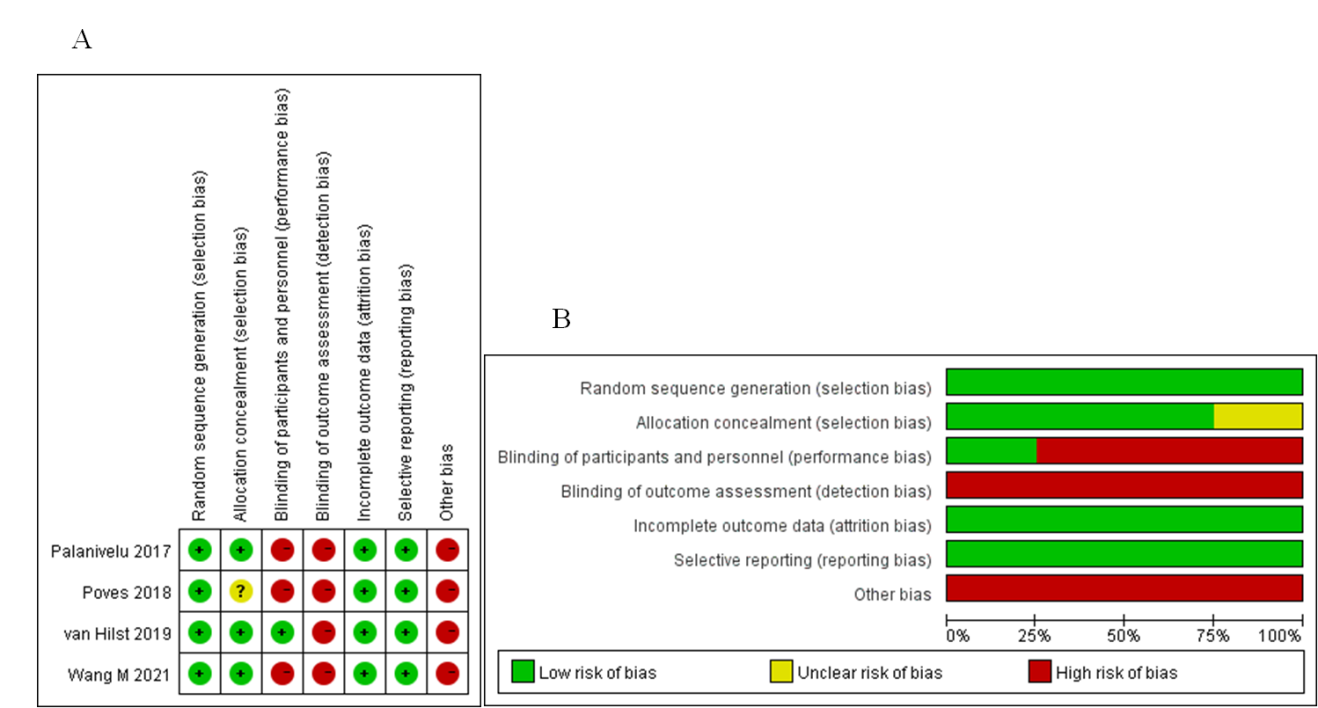


**Supplementary Figure 2.** Risk of bias summary (A) and risk of bias graph (B).


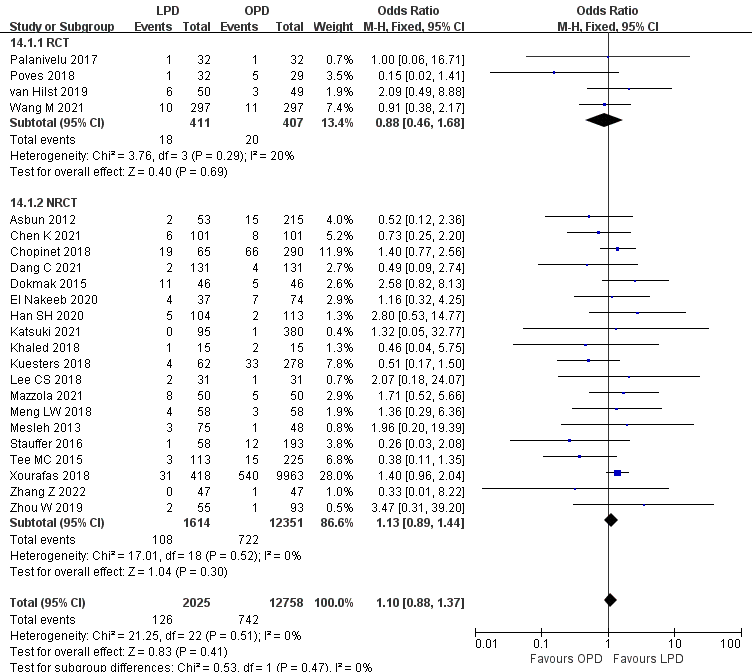


**Supplementary Figure 3.** Forest plot of comparison between LPD and OPD on reoperation.


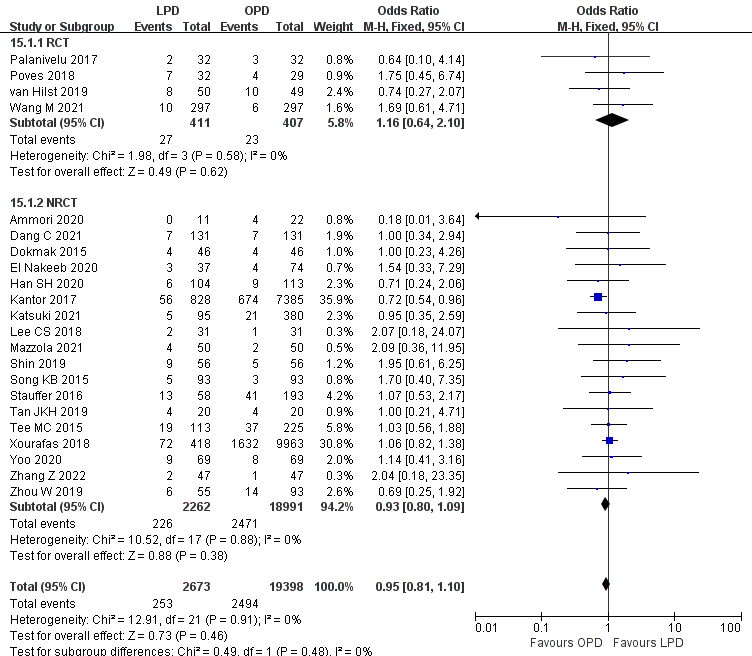


**Supplementary Figure 4.** Forest plot of comparison between LPD and OPD on unplanned readmission.


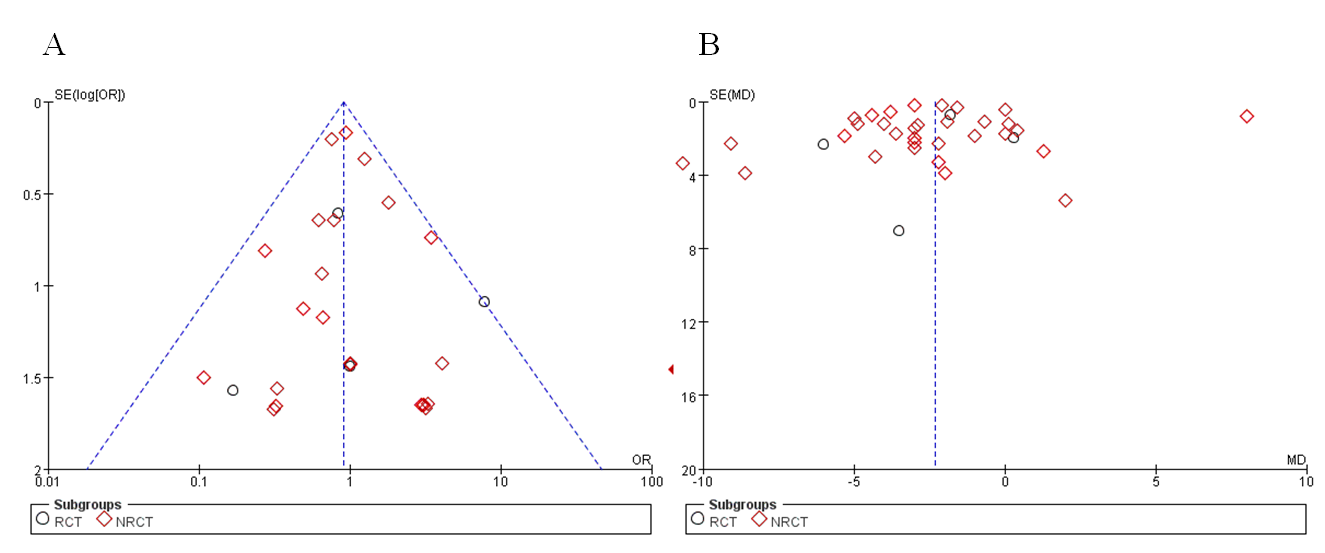


**Supplementary Figure 5.** Funnel plots of postoperative mortality (A) and hospital stay (B) for assessing publication bias.
